# Clinical Features of Cellular Senescence Pathways in Severe Asthma

**DOI:** 10.1111/all.70416

**Published:** 2026-06-18

**Authors:** Woo‐Jung Song, Nazanin Zounemat Kermani, Ali Versi, Stephany Sánchez‐Ovando, Jodie Louise Simpson, Peter A. Wark, Katherine Joanne Baines, Sven‐Erik Dahlén, Ratko Djukanovic, Yike Guo, Ian M. Adcock, Kian Fan Chung, the U‐BIOPRED Study Group, Mahmoud. I. Abdel‐Aziz, Ian M. Adcock, Lars I. Andersson, Charles Auffray, Yusef E. Badi, Per Bakke, Aruna T. Bansal, Frederic Baribaud, Stewart A. Bates, Elisabeth H. Bel, Jeanette Bigler, Bo Billing, Hans Bisgaard, Michael J. Boedigheimer, Klaus Bønnelykke, Joost Brandsma, Paul Brinkman, Enrica Bucchioni, Dominic Burg, Andrew Bush, Massimo Caruso, Romanas Chaleckis, Pascal Chanez, Kian Fan Chung, T. Checa, Chris H. Compton, Julie Corfield, Danen Cunoosamy, Arnaldo D'Amico, Barbro Dahlén, Sven‐Erik Dahlén, Bertrand De Meulder, Ratko Djukanovic, Veit J. Erpenbeck, Damijan Erzen, K. Fichtner, Louise J. Fleming, Elena Formaggio, Stephen J. Fowler, Urs Frey, Martina Gahlemann, Thomas Geiser, Victoria Goss, Yike Guo, Simone Hashimoto, John Haughney, Gunilla Hedlin, Pieter‐Paul W. Hekking, Timothy Higenbottam, Jens M. Hohlfeld, Cecile Holweg, Ildiko Horvath, Peter Howarth, Anna J. James, Richard G. Knowles, Johan Kolmert, Jon Konradsen, Norbert Krug, Nikos Lazarinis, C.‐X. Li, Matthew J. Loza, Rene Lutter, Alexander Manta, Sarah Masefield, Anke H. Maitland‐van der Zee, John G. Matthews, Alexander Mazein, Roelinde J. M. Middelveld, Montserrat Miralpeix, Paolo Montuschi, Jacek Musial, Sharon Mumby, David Myles, Björn Nordlund, Ioannis Pandis, Stelios Pavlidis, Anthony Postle, P. Powel, G. Praticò, M. Puig Valls, N. Rao, Stacey Reinke, John Riley, Amanda Roberts, Graham Roberts, Anthony Rowe, Thomas Sandström, Jim P. R. Schofield, Wolfgang Seibold, Dominic E. Shaw, R. Sigmund, Florian Singer, Paul J. Skipp, M. Smicker, Ana R. Sousa, Maria Sparreman‐Mikus, Peter J. Sterk, Marika Ström, Kai Sun, Bob Thornton, Mohib Uddin, Ali Versi, Jorgen Vestbo, Nadja H. Vissing, Scott S. Wagers, Åsa Wheelock, Craig E. Wheelock, Susan J. Wilson, Valentyna Yasinska, Nazanin Zounemat Kermani

**Affiliations:** ^1^ Department of Allergy and Clinical Immunology, Asan Medical Center University of Ulsan College of Medicine Seoul Korea; ^2^ National Heart and Lung Institute Imperial College London London UK; ^3^ Department of Computing, Data Science Institute Imperial College London London UK; ^4^ Priority Research Centre for Healthy Lungs, Faculty of Health and Medicine University of Newcastle Newcastle New South Wales Australia; ^5^ Allergy, Immunology and Respiratory Medicine Alfred Health Melbourne Victoria Australia; ^6^ Centre for Allergy Research Karolinska Institute Stockholm Sweden; ^7^ Faculty of Medicine Southampton University Southampton UK; ^8^ NIHR Southampton Respiratory Biomedical Research Unit University Hospital Southampton Southampton UK

**Keywords:** cellular senescence, nasal polyps, p53, senescence‐associated secreted phenotype, severe asthma

## Abstract

**Background:**

Asthma severity increases with age, suggesting a role for accelerated biological ageing. We hypothesised that cellular senescence pathways such as the senescence‐associated secretory pathway (SASP) and the p53‐cellular senescence pathway are enriched in the airways of patients with severe asthma.

**Methods:**

We utilised transcriptomic data from the U‐BIOPRED cohort to analyse enrichment scores (ES) of p53 and SASP pathways in different airway compartments using gene set variation analysis. Findings in bronchial biopsies were validated in the independent NOVA cohort. We examined associations between senescence ES, clinical parameters and other asthma‐related gene signatures. Functional clusters of the SASP gene set were also explored.

**Results:**

In the U‐BIOPRED cohort, p53 and SASP ES were significantly elevated in bronchial biopsies of severe asthmatics compared to mild‐to‐moderate asthmatics and healthy volunteers, with SASP enrichment validated in the NOVA cohort. In bronchial biopsies, higher senescence ES correlated with frequent exacerbations, oral corticosteroid use, comorbid nasal polyps and lower FEV1%. No significant enrichment was found in other airway samples according to asthma severity. In nasal brushings, SASP ES was significantly higher in participants with comorbid nasal polyps. A distinct SASP functional cluster related to lung injury and repair (Cluster 2) was strongly associated with clinical severity and nasal polyps. Senescence signatures correlated positively with oxidative phosphorylation and macrophage activation signatures, but not with eosinophil signatures.

**Conclusions:**

Cellular senescence pathways are enriched in severe asthmatic bronchial tissues and correlate with disease severity, remodelling and nasal polyps. These findings warrant further investigation into their therapeutic implications.

**Trial Registration:**

NCT01982162

## Graphical abstract

In bronchial biopsies, SASP and p53 pathway enrichment scores are elevated in severe asthma versus non‐severe asthma and healthy controls. SASP enrichment is validated in the independent NOVA cohort. SASP enrichment is also elevated in nasal brushings of participants with nasal polyps, independently of asthma status. Senescence pathway enrichment associates with exacerbations, daily OCS, lower post‐BD FEV1% and macrophage/OXPHOS signatures, but not with Th2/eosinophil signatures, supporting a non‐T2 mechanism. FeNO, fractional exhaled nitric oxide; FEV1, forced expiratory volume in 1 s; NOVA, Newcastle severe asthma cohort; OCS, oral corticosteroid; OXPHOS, oxidative phosphorylation; p53, tumour protein 53; post‐BD, post‐bronchodilator; SASP, senescence‐associated secretory phenotype; Th2, T helper 2; U‐BIOPRED, Unbiased Biomarkers for the Prediction of Respiratory Disease Outcomes.

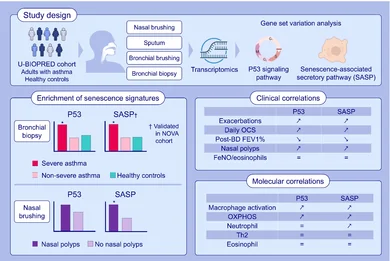

## 1 Introduction

Asthma is common across the lifespan but can present with a greater degree of severity in older adults [1, 2, 3]. Clusters of older, more severe asthmatics with greater symptom burden, worse lung function and more exacerbations have been observed [4, 5, 6, 7]. This age‐related increase in asthma severity has been associated with greater lung function decline and more comorbidities, with the potential contribution of air pollution, cigarette smoke or infection [8, 9, 10]. The distinct features characterising these severe asthma clusters may be consequential to cellular senescence leading to an accelerated airway ageing.

Cellular senescence can be induced by repeated exposure to stressful triggers and has been characterised by prolonged and irreversible cell‐cycle arrest with a senescence‐associated secreted phenotype (SASP) [11]. Cellular senescence has been implicated as a potentially important driver of age‐related chronic inflammatory lung diseases such as chronic obstructive pulmonary disease or idiopathic pulmonary fibrosis (IPF) [12, 13]. In asthma, markers of cellular senescence, including the transcription factors p53 and p21, and SASP factors, have been reported in bronchial fibroblasts and epithelial cells, sputum and blood cells [14, 15, 16, 17, 18, 19, 20]. A recent transcriptomic analysis of publicly available datasets has reported differentially expressed cellular senescence‐related genes in severe asthma; however, it was limited in its ability to link transcriptomic findings to individual‐level clinical characteristics [18]. To date, a comprehensive assessment of senescence pathways across different airway compartments in clinical cohorts is lacking. We therefore hypothesised that cellular senescence pathways are enriched in the airways of patients with severe asthma and that these drive the severity of the disease.

In this study, we examined the enrichment of two major cellular senescence pathways representing cell‐cycle arrest and secretory phenotypes, the p53 signalling pathway and SASP [11], in different airway samples. These included nasal brushings, bronchial brushings, bronchial biopsies and induced sputum‐derived cells from asthmatics in the Unbiased Biomarkers for the Prediction of Respiratory Disease Outcomes (U‐BIOPRED) cohort [21, 22]. Additionally, the Australian Priority Research Centre for Healthy Lungs—NOVocastrian Asthma cohort (NOVA) [23] was used as an independent validation cohort.

## 2 Methods

### 2.1 Participants

This study analysed severe asthmatics (SA), mild‐to‐moderate asthmatics (MMA) and non‐smoking healthy volunteers (HV) in the U‐BIOPRED cohort, as previously described [22]. Details of the NOVA cohort (42 SA, 16 MMA and 13 HV) have been reported elsewhere [23]. All participants were managed according to severe asthma management guidelines; adherence to prescribed therapy was systematically assessed at each study visit, and non‐adherent patients were excluded from the severe asthma category, as described in the original cohort publications [22, 23]. The studies were approved by the local Ethics Committees, and all participants gave signed informed consent.

### 2.2 Participant Assessment

Participants underwent baseline assessment for demographic and clinical parameters including age, sex, body mass index, smoking history, age of asthma onset, asthma duration, allergic comorbidities, the number of asthma exacerbations during the past year, oral corticosteroid (OCS) use, spirometry, serum total IgE, blood differential cell counts, induced sputum cell counts, fractional exhaled nitric oxide (FeNO) and serum periostin following a standardised operating procedure. Atopic status was confirmed by skin prick testing or specific immunoglobulin E measurements to six common aeroallergens. The presence of nasal polyps was defined by physician‐diagnosed history.

### 2.3 Microarray Analysis of Transcriptome

Gene expression was profiled using U133Plus 2.0 microarray (Affymetrix, Santa Clara, CA) on total RNA extracted from induced sputum cells, bronchial brushings, bronchial biopsies and nasal brushings as previously described [21]. Only samples meeting stringent RNA quality criteria (RNA Integrity Number [RIN] ≥ 6.0, 260/280 ratio 1.8–2.1) and minimum quantity thresholds were included in the microarray analysis, ensuring comparable data quality across all airway sample types [21, 24]. The transcriptomic data were assessed by multiarray average normalisation using the Almac Pipeline and Pre‐processing Toolbox (Almac, Craigavon, UK).

### 2.4 Expression of Signatures by Gene Set Variation Analysis

We evaluated gene expression related to cellular senescence using gene set variation analysis (GSVA). GSVA calculates sample‐wise enrichment scores (ES), irrespective of any group label [25]. Based on the consensus framework for defining cellular senescence proposed by Gorgoulis et al. [11], we selected a priori two gene signatures representing the two complementary hallmarks of cellular senescence: the p53 signalling pathway (representing cell‐cycle arrest) and the SASP. The p53 signalling gene set was derived from the KEGG database (hsa04115), and the SASP gene set from the Reactome database (R‐HSA‐2559582), both of which have been used to characterise senescent transcriptional programmes [26, 27]. The full gene lists are provided in Table S1. We also explored gene signatures related to asthma mechanisms or pathways (Table S2) and analysed their ES correlations with those of p53 and SASP.

### 2.5 Functional Clusters of SASP Gene Set

Due to the large number of genes in the SASP gene set and the functional heterogeneity of proteins that these genes encode, we used protein–protein interaction data provided by STRINGDB (https://string‐db.org) and *K*‐means clustering to group the proteins into highly interactive and connected subgroups. ES for each cluster was calculated by GSVA.

### 2.6 Data Analysis

All datasets were curated and stored in tranSMART, an open‐source knowledge management platform for sharing research data, supported by the European Translational Information and Knowledge Management Services project (www.etriks.org) [28]. Variables with skewed distributions were log‐transformed. One‐way ANOVA and Tukey's multiple comparison tests were utilised to examine differences across 3 groups (SA, MMA and HV). To quantify the magnitude of between‐group differences, Cohen's *d* was calculated as the difference in group means divided by the pooled standard deviation. Effect sizes were interpreted according to conventional thresholds (small, |*d*| ≥ 0.2; medium, |*d*| ≥ 0.5; large, |*d*| ≥ 0.8). To complement continuous‐score analyses, participants were dichotomised at the cohort‐wide median of each ES and assigned to one of four quadrants (p53‐low/SASP‐low, p53‐low/SASP‐high, p53‐high/SASP‐low, p53‐high/SASP‐high). Differences in quadrant distribution across cohorts were assessed using Pearson's chi‐square test.

Linear regression analyses were used to examine the relationships of the gene set ES with other parameters. Multivariate linear regressions were conducted to examine whether the relationships were independent of confounders. Spearman's correlations of ES between different signatures were calculated. All statistical analyses were performed using Stata version 18.0 (StataCorp, College Station, TX, USA), with *p*‐values < 0.05 considered statistically significant. For exploratory analyses involving screening of multiple gene signatures, *p*‐values were adjusted using the Benjamini‐Hochberg method to control the false discovery rate (FDR) and only FDR‐adjusted *p*‐values < 0.05 were considered statistically significant.

## 3 Results

### 3.1 SASP and p53 Pathway Enrichment in Bronchial Biopsies of Severe Asthma

**FIGURE 1:**
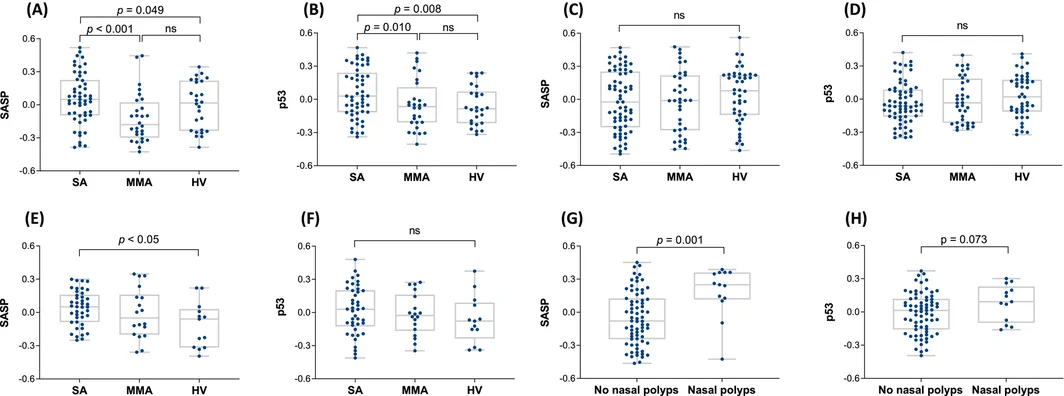
Comparison of SASP and p53 signalling pathway signatures using gene set enrichment scores (ES). (A) SASP and (B) p53 signalling pathway enrichment in bronchial biopsies obtained from the U‐BIOPRED cohort, according to asthma status and severity. No enrichment of (C) SASP and (D) p53 signalling pathways was observed in bronchial brushings. (E) Validation of significantly increased SASP, but not (F) p53 signalling pathway enrichment in bronchial biopsies from the Australian NOVA cohort. Enrichment of (G) SASP and (H) p53 signalling pathways in nasal brushing samples according to the presence or absence of nasal polyps. HV, healthy volunteers; MMA, mild‐to‐moderate asthmatics; ns, not significant (*p* > 0.05); SA, severe asthmatics; SASP, senescence‐associated secretory phenotype.

In the U‐BIOPRED cohort, the ES for both the SASP and p53 signalling pathways was significantly elevated in the bronchial biopsies from participants with SA compared to those with MMA and HV (Figure 1A,B). In contrast, no significant differences in these pathway ES were observed across the groups in bronchial brushings (Figure 1C,D), induced sputum or nasal brushings (data not shown). Consequently, subsequent analyses focused specifically on bronchial biopsies within the U‐BIOPRED cohort. The baseline characteristics of the 107 participants who provided bronchial biopsies (53 SA, 28 MMA and 26 HV) are summarised in Table 1. Importantly, the increased enrichment of the SASP pathway, but not the p53 pathway, in bronchial biopsies of patients with SA compared to HVs was validated in the independent NOVA cohort (42 SA, 18 MMA and 13 HV; Figure 1E,F).

**TABLE 1:** Baseline characteristics of study participants with bronchial biopsies.

	SA	MMA	HV	*p*
Participant, *n*	53	28	26	
Age (years)	52 (43–59)	42.5 (28.5–50)	37 (25–53.2)	0.001
Female, *n* (%)	28 (52.8%)	16 (57.1%)	10 (38.5%)	0.347
BMI (kg/m^2^)	28.4 (25.2–33.8)	26.0 (22.7–29.0)	24.4 (22.7–27.3)	< 0.001
Smoking status, *n* (%)				
Current smoker	7 (13.2%)	0 (0%)	0 (0%)	0.031
Former smoker	12 (22.6%)	3 (10.7%)	4 (15.4%)	
Never smoker	34 (64.2%)	25 (89.3%)	22 (84.6%)	
Smoking (pack‐years)	0 (0–5)	0 (0–0)	0 (0–0)	
Asthma onset age (years)	13 (2–43)	13 (7–25)	na	0.604
Asthma duration (years)	25.5 (15.5–42.5)	24.5 (15–33)	na	0.465
Allergic rhinitis, *n* (%)	27 (50.9%)	12 (44.4%)	1 (3.8%)	0.001
Nasal polyps, *n* (%)	19 (36.5%)	0 (0%)	1 (3.9%)	< 0.001
Maintenance OCS use, *n* (%)	19 (36.5%)	0 (0%)	0 (0%)	< 0.001
Exacerbation frequency in the previous year	9 (2–11)	1 (1–1)	na	< 0.001
FEV1% pred (%)	72.2 (55.3–85.2)	92.4 (77.3–103.8)	99.0 (89.6–110.4)	< 0.001
FVC% pred (%)	90.5 (81.4–101.1)	109.1 (92.1–116.6)	109.0 (96.8–112.7)	< 0.001
FEV1/FVC ratio (%)	66.8 (55.2–75.6)	72.9 (68.7–81.2)	79.6 (75.3–82.3)	< 0.001
Post‐BD FEV1% pred (%)	79.79 (67.4–90.9)	102.8 (88.7–109.2)	na	< 0.001
FeNO (ppb)	24.3 (15.8–44.5)	20.5 (13.3–50.0)	24.0 (15.0–31.0)	0.606
Total IgE (IU/mL)	124.0 (37.5–415.0)	86.3 (44.0–152.0)	23.0 (7.0–96.2)	0.001
Serum periostin (ng/mL)	45.6 (40.6–51.6)	43.6 (38.2–51.8)	55.1 (45.2–60.8)	0.021
Blood eosinophil (×10^9^/L)	0.2 (0.1–0.3)	0.17 (0.1–0.3)	0.1 (0.1–0.15)	0.004
Blood neutrophil (×10^9^/L)	4.9 (3.6–6.6)	3.7 (2.7–4.7)	3.0 (2.1–3.9)	< 0.001

*Note:* Variables are described in %, or median (interquartile range 25%–75%). The chi‐squared test was used for computing the *p* value of categorical variables. Normality of numerical variables was examined by the Shapiro–Wilk test. Mann–Whitney *U* test was used for non‐normally distributed numerical variables and pairwise Student's *t*‐test for normally distributed numerical variables.

Abbreviations: BMI, body mass index; FeNO, fractional exhaled nitric oxide; FEV1, forced expiratory volume in 1 s; FVC, forced vital capacity; HV, healthy volunteer; MMA, mild‐to‐moderate asthma; na, not applicable; OCS, oral corticosteroids; post‐BD, post‐bronchodilator; SA, severe asthma.

The effect sizes (Cohen's *d*) in the U‐BIOPRED cohort were moderate‐to‐large for SASP (SA vs. MMA, *d* = 0.76) and small‐to‐moderate for p53 (SA vs. MMA, *d* = 0.46; SA vs. HV, *d* = 0.53). In the NOVA cohort, SASP showed a similar medium‐to‐large effect (SA vs. HV, *d* = 0.77), whereas p53 showed a small, non‐significant effect (*d* = 0.40, *p* = 0.23), despite a consistent directional trend (SA > MMA > HV).

### 3.2 Co‐Enrichment of p53 and SASP Pathways in Severe Asthma

**FIGURE 2:**
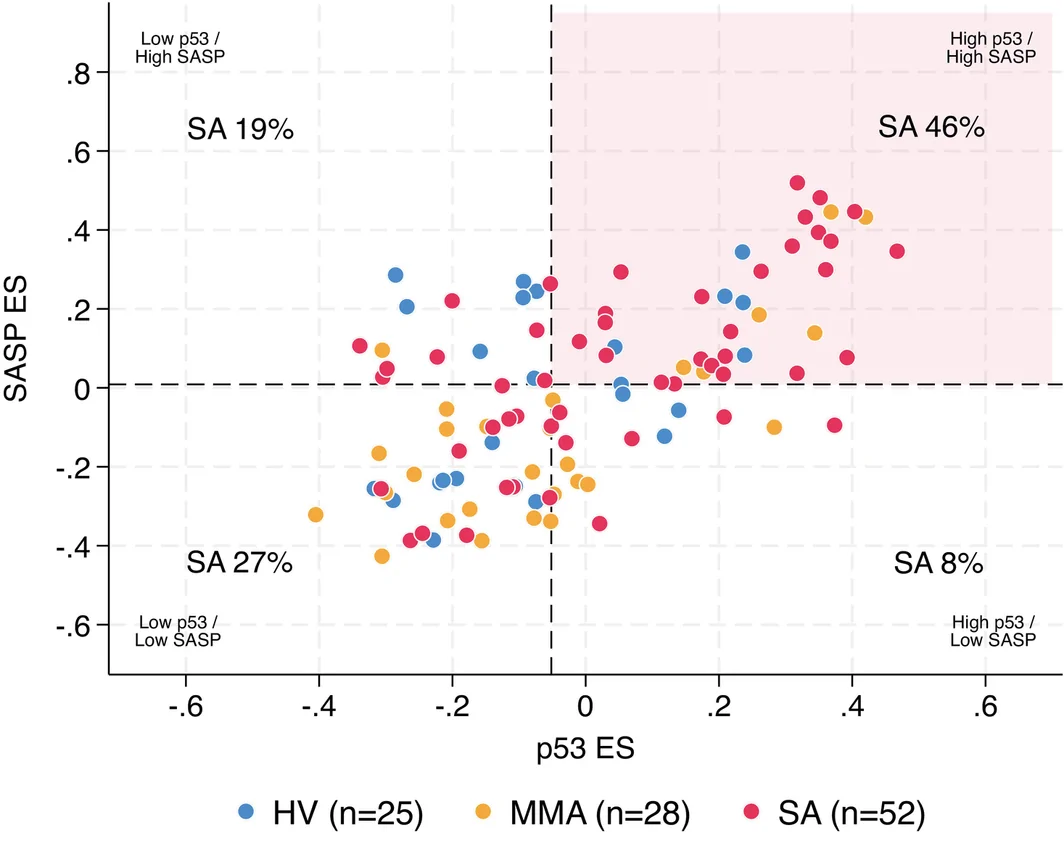
Co‐enrichment of p53 and SASP signalling pathways in bronchial biopsies from the U‐BIOPRED cohort. Scatter plot of p53 versus SASP enrichment scores (ES) for individuals according to asthma status and severity. Dashed lines indicate the cohort‐wide median of each score. The shaded area corresponds to the dual‐high (p53‐high/SASP‐high) quadrant. Numbers within each quadrant denote the proportion of severe asthmatics (SA) assigned to that quadrant. ES, enrichment score; HV, healthy volunteers; MMA, mild‐to‐moderate asthmatics; SA, severe asthmatics; SASP, senescence‐associated secretory phenotype.

To complement the continuous‐score analyses, the U‐BIOPRED participants were dichotomised at the cohort‐wide medians of each score and assigned to one of four quadrants (Figure 2). Quadrant distribution differed significantly across SA, MMA and HV (*p* = 0.008). Severe asthmatics were enriched in the dual‐high (p53‐high/SASP‐high) quadrant—46% (24/52) of SA, versus 21% (6/28) of MMA and 24% (6/25) of HV (*p* = 0.039 for dual‐high vs. other quadrants).

### 3.3 Associations With Demographics and Asthma Outcomes

We next examined whether SASP and p53 pathway enrichment scores were associated with clinical outcomes. Univariate linear regression analyses revealed that higher ES of both the SASP and p53 pathways in the bronchial biopsies were significantly associated with indicators of disease severity, including greater asthma exacerbation frequency in the previous year, daily maintenance OCS use, comorbid nasal polyps and poorer lung function (lower FEV1% predicted and post‐BD FEV1%) (Table 2).

**TABLE 2:** Linear regression between baseline parameters and the cellular senescence pathway gene set enrichment score (ES) in bronchial biopsies of patients with asthma.

Parameter	p53 signalling pathway gene set ES		SASP gene set ES	
	Regression coefficient (95% CI)	*p*	Regression coefficient (95% CI)	*p*
Age (years)	0.001 (−0.003 to 0.005)	0.578	0.002 (−0.002 to 0.006)	0.404
Male (vs. female)	0.085 (−0.015 to 0.186)	0.096	0.092 (−0.015 to 0.199)	0.090
BMI (kg/m^2^)	−0.005 (−0.014 to 0.004)	0.281	−0.001 (−0.010 to 0.009)	0.849
Smoking (pack‐year)	−0.002 (−0.008 to 0.005)	0.633	0.003 (−0.004 to 0.010)	0.439
Asthma onset age (years)	0.001 (−0.002 to 0.004)	0.423	0.003 (0.0002 to 0.006)	0.034
Exacerbations in the previous year (number)^a^	0.061 (0.017 to 0.106)	0.008	0.090 (0.045 to 0.135)	< 0.001
Maintenance OCS use (vs. no)	0.131 (0.027 to 0.235)	0.014	0.181 (0.073 to 0.288)	0.001
Nasal polyp (vs. no)	0.228 (0.118 to 0.337)	< 0.001	0.206 (0.087 to 0.325)	0.001
FEV1% pred (%)^a^	−0.189 (−0.372 to −0.008)	0.041	−0.255 (−0.445 to −0.065)	0.009
FVC% pred (%)^a^	−0.279 (−0.530 to −0.028)	0.030	−0.262 (−0.530 to 0.006)	0.056
FEV1/FVC ratio (%)	−0.210 (−0.636 to 0.217)	0.331	−0.318 (−0.603 to −0.033)	0.029
Post‐BD FEV1% pred (%)	−0.283 (−0.469 to −0.098)	0.003	−0.319 (−0.513 to −0.125)	0.002
FeNO (ppb)^a^	0.027 (−0.042 to 0.095)	0.445	0.008 (−0.065 to 0.080)	0.836
Serum total IgE (IU/mL)^a^	0.014 (−0.018 to 0.047)	0.374	0.001 (−0.034 to 0.035)	0.977
Serum periostin (ng/mL)^a^	0.394 (0.163 to 0.626)	0.001	0.339 (0.083 to 0.595)	0.010
Blood eosinophil (%)^a^	0.032 (−0.037 to 0.101)	0.356	0.030 (−0.043 to 0.102)	0.416
Blood neutrophil (%)^a^	0.062 (−0.058 to 0.181)	0.308	0.059 (−0.068 to 0.186)	0.355

*Note:* Values indicate regression coefficients (95% confidence intervals) and *p* values from univariate linear regression analyses.

Abbreviations: 95% CI, 95% confidence interval; BMI, body mass index; ES, enrichment score; FeNO, fractional exhaled nitric oxide; FEV1, forced expiratory volume in 1 s; FVC, forced vital capacity; OCS, oral corticosteroids; post‐BD, post‐bronchodilator; SASP, senescence‐associated secretory phenotype.

^a^ Variable log‐transformed to normalise distribution.

Neither SASP nor p53 scores were correlated with chronological age, although SASP ES were associated with the age of asthma onset (Table 2). This absence of correlation was consistent within each clinical group: MMA (*p* = 0.135 and *p* = 0.213) and SA (*p* = 0.438 and *p* = 0.422, for SASP and p53 respectively).

Among the examined inflammatory markers, only serum periostin levels showed a significant association with the ES of both the SASP and p53 pathways. These associations with clinical parameters remained statistically significant in multivariate regression analyses after adjusting for potential confounders, including age, sex, smoking pack‐year and body mass index (Table 3A).

**TABLE 3:** Multivariate linear regression between clinical parameters and the cellular senescence pathway gene set enrichment score (ES) in (A) bronchial biopsies and (B) nasal brushing.

(A) Bronchial biopsy	p53 signalling pathway gene set ES		SASP gene set ES	
Parameter	Regression coefficient (95% CI)^b^	*p*	Regression coefficient (95% CI)^b^	*p*
Asthma onset age (years)	0.001 (−0.002 to 0.005)	0.403	0.003 (0.000 to 0.007)	0.047
Exacerbations in the previous year (number)^a^	0.075 (0.029 to 0.120)	0.002	0.094 (0.047 to 0.141)	< 0.001
Maintenance OCS use (vs. no)	0.150 (0.039 to 0.261)	0.009	0.194 (0.079 to 0.310)	0.001
Nasal polyp (vs. no)	0.240 (0.120 to 0.360)	< 0.001	0.195 (0.061 to 0.328)	0.005
Post‐BD FEV1% pred (%)	−0.284 (−0.495 to −0.073)	0.009	−0.287 (−0.510 to −0.063)	0.013
Serum periostin (ng/mL)^a^	0.370 (0.123 to 0.616)	0.004	0.303 (0.029 to 0.577)	0.031

(B) Nasal brushing	p53 signalling pathway gene set ES		SASP gene set ES	
Parameter	Regression coefficient (95% CI)^c^	*p*	Regression coefficient (95% CI)^c^	*p*
Age (years)	0.0005 (−0.003 to 0.004)	0.758	0.002 (−0.003 to 0.006)	0.427
Male (vs. female)	0.070 (−0.015 to 0.154)	0.105	0.052 (−0.062 to 0.165)	0.365
Ever smoking (vs. never)	−0.046 (−0.145 to 0.054)	0.363	−0.023 (−0.157 to 0.110)	0.728
Asthma (vs. no)	0.090 (−0.008 to 0.186)	0.070	0.115 (−0.015 to 0.245)	0.083
Nasal polyp (vs. no)	0.065 (−0.055 to 0.184)	0.283	0.194 (0.035 to 0.354)	0.018

*Note:* Values indicate regression coefficients (95% confidence intervals).

Abbreviations: 95% CI, 95% confidence interval; ES, enrichment score; FEV1, forced expiratory volume in 1 s; OCS, oral corticosteroids; post‐BD, post‐bronchodilator; SASP, senescence‐associated secretory phenotype.

^a^ Variable log‐transformed to normalise distribution.

^b^ Adjusted for age, sex, smoking pack‐year and body mass index.

^c^ Adjusted for age, sex, smoking, asthma and nasal polyps.

Among patients with SA, neither p53 nor SASP ES differed significantly between those on daily maintenance OCS and those not (Table S3). Importantly, the associations with exacerbation frequency, nasal polyps, post‐BD FEV1% and serum periostin levels remained significant after further adjustment for maintenance OCS use (Table S4).

We next examined the clinical correlates of the quadrant classification (p53/SASP) among patients with asthma (*n* = 80). Consistent with the associations above, the p53‐high/SASP‐high quadrant (*n* = 30) exhibited the lowest post‐BD FEV1% predicted (median 79.8% [interquartile range (IQR) 56.9%–94.0%], *p* = 0.028), the highest exacerbation count in the previous year (median 9.0 [IQR 1.2–11.0], *p* = 0.008), the highest prevalence of nasal polyps (45%, *p* = 0.006) and the highest maintenance OCS use (47%, *p* = 0.042), while demographic features (age, sex, BMI, smoking history) and type‐2 inflammatory markers (eosinophils, FeNO, total IgE, periostin) did not differ significantly across quadrants. The number of subjects in the discordant quadrants was small (p53‐low/SASP‐high, *n* = 11; p53‐high/SASP‐low, *n* = 6); however, they appeared to represent an intermediate phenotype: their maintenance OCS use (36% and 50%, respectively) was higher than that of the p53‐low/SASP‐low quadrant (15%) and comparable to that of the p53‐high/SASP‐high quadrant (47%), while their exacerbation frequency was moderately elevated (median 3.0 and 2.0, respectively) (Table S5).

### 3.4 Associations With Nasal Polyps in Nasal Brushings

Given the significant associations of both SASP and p53 pathway ES in bronchial biopsies of participants with comorbid nasal polyps, we further examined these associations in nasal brushings, which were available for all participant groups including HVs. Our results confirmed that in the nasal brushings, the SASP ES was significantly elevated in participants with nasal polyps compared to those without (Figure 1G, *p* < 0.001). In contrast, the p53 pathway ES did not show a significant association with the presence of nasal polyps (Figure 1H, *p* = 0.073). Importantly, in multivariate regression analyses, the SASP ES in nasal brushings remained positively associated with the presence of nasal polyps, independent of age, sex, smoking history and the presence of asthma (Table 3B).

### 3.5 Exploration Using Functional Clusters of SASP Gene Set

**FIGURE 3:**
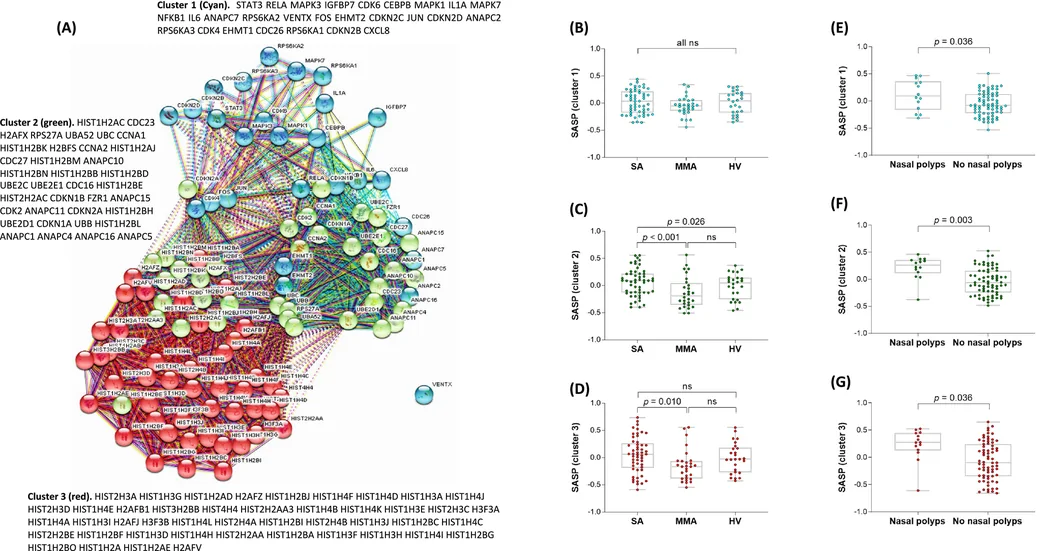
Identification of three SASP gene clusters using protein–protein interaction (PPI) networks (https://string‐db.org). (A) PPI network analysis of SASP genes revealed three highly interactive and connected clusters. Representative genes for each cluster are shown (Cluster 1: Cyan; Cluster 2: Green; Cluster 3: Red). Gene enrichment score (ES) comparisons in bronchial biopsies according to asthma severity for (B) Cluster 1, (C) Cluster 2 and (D) Cluster 3. ES comparisons in nasal brushings based on the presence or absence of nasal polyps for (E) Cluster 1, (F) Cluster 2 and (G) Cluster 3. HV, healthy volunteers; MMA, mild‐to‐moderate asthmatics; ns, not significant (*p* > 0.05); SA, severe asthmatics.

K‐means clustering of SASP gene interaction data identified three functionally distinct gene clusters (Figure 3A). Cluster 1 was characterised by enrichment in pathways involved in oxidative stress‐induced senescence, ribosomal S6 kinase (RSK) activation and IL‐6 signalling. These genes are implicated in airway inflammation, therapeutic responses (e.g., to omalizumab and PI3K inhibitors) and TH2/TH17‐high asthma endotypes. Cluster 2 included genes such as *HIST, CDKN1B* and *CCNA2*, which were related to T cell regulation, lung injury, repair processes and lung cancer biology. Cluster 3, dominated by HIST family genes, was linked to steroid‐resistance, environmentally influenced immune responses and pathways of airway remodelling (detailed in Online Repository).

In bronchial biopsies, the ES of Cluster 1 (Figure 3B) did not differ significantly across the groups. However, the ES of Cluster 2 (Figure 3C) and Cluster 3 (Figure 3D) mirrored the overall SASP pathway trend across asthma severities, with Cluster 2 displaying the most pronounced differences. Linear regression analyses revealed that SASP Cluster 2 exhibited stronger associations with post‐BD FEV1% and comorbid nasal polyps compared to the other clusters (Table S6A).

In nasal brushings, all three clusters (Figure 3E–G) showed significantly higher ES in participants with nasal polyps compared to those without. Similar to bronchial biopsies, the inter‐group difference was greater for Cluster 2 (Figure 3F), which was also validated in multivariate linear regression analyses (Table S6B).

### 3.6 Relevance of the p53 and SASP Pathways to Different Asthma Gene Signatures

To explore molecular pathways underlying cellular senescence in asthma and nasal polyps, we correlated the p53 and SASP ES with ES of various asthma‐related gene signatures (Table 4 and Table S2) in the bronchial biopsies and nasal brushings. In both the bronchial biopsies and nasal brushings, the ES of macrophage activation signatures (LPS‐stimulated_macrophage, Macrophage.GM_CSF.TNFa.HS.IVS.UP and IFNG‐stimulated_macrophage) and oxidative phosphorylation (OXPHOS) showed more significant positive correlations with p53 and SASP ESs compared to the other asthma‐related signatures. Notably, SASP Cluster 2 exhibited the most robust correlation with OXPHOS in nasal brushings (*r* = 0.78, *p* < 0.001; Table 4).

**TABLE 4:** Correlations between the p53, SASP and SASP cluster enrichment score (ES) and asthma*‐*related signature ES in bronchial biopsy and nasal brushing transcriptomics data of patients with asthma.

Asthma‐related signature	Bronchial biopsy					Nasal brushing				
	p53 ES	SASP ES	SASP cluster ES			p53 ES	SASP ES	SASP cluster ES		
			Cluster 1	Cluster 2	Cluster 3			Cluster 1	Cluster 2	Cluster 3
LPS‐stimulated_macrophage	0.50 (***)	0.64 (***)	0.52 (***)	0.62 (***)	0.49 (***)	0.64 (***)	0.67 (***)	0.66 (***)	0.54 (***)	0.58 (***)
Macrophage.GM_CSF.TNFa.HS.IVS.UP	0.45 (***)	0.44 (***)	0.51 (***)	0.41 (***)	0.28 (ns)	0.63 (***)	0.57 (***)	0.65 (***)	0.43 (***)	0.49 (***)
OXPHOS	0.34 (**)	0.58 (***)	0.2 (ns)	0.55 (***)	0.60 (***)	0.44 (***)	0.69 (***)	0.16 (ns)	0.78 (***)	0.63 (***)
IFNG‐stimulated_macrophage	0.30 (*)	0.46 (***)	0.52 (***)	0.38 (**)	0.31 (*)	0.44 (***)	0.49 (***)	0.74 (***)	0.29 (ns)	0.36 (*)
CD4	0.28 (ns)	0.49 (***)	0.43 (***)	0.42 (***)	0.34 (**)	0.33 (*)	0.35 (*)	0.64 (***)	0.14 (ns)	0.22 (ns)
IL17a	0.15 (ns)	0.09 (ns)	0.21 (ns)	0.04 (ns)	−0.01 (ns)	0.21 (ns)	0.19 (ns)	0.53 (***)	0.07 (ns)	0.08 (ns)
IL‐13_stimulated HBEC	0.11 (ns)	0.11 (ns)	0.07 (ns)	0.1 (ns)	0.09 (ns)	0.24 (ns)	0.22 (ns)	0.27 (ns)	0.13 (ns)	0.2 (ns)
Inflammasome	0.09 (ns)	0.15 (ns)	0.31 (*)	0.1 (ns)	0.12 (ns)	0.15 (ns)	0.35 (*)	0.61 (***)	0.08 (ns)	0.22 (ns)
Macrophage.GM_CSF.IL4	0.08 (ns)	0.1 (ns)	0.17 (ns)	0.04 (ns)	0.16 (ns)	0.18 (ns)	0.24 (ns)	0.46 (***)	0.12 (ns)	0.2 (ns)
Mast cell	0 (ns)	−0.11 (ns)	0.06 (ns)	−0.12 (ns)	−0.12 (ns)	0.23 (ns)	0.29 (ns)	0.23 (ns)	0.2 (ns)	0.39 (*)
Neutrophil	−0.04 (ns)	0.29 (*)	0.39 (***)	0.09 (ns)	0.15 (ns)	0.17 (ns)	0.33 (*)	0.66 (***)	0.02 (ns)	0.18 (ns)
ILC1_cell	−0.08 (ns)	−0.02 (ns)	0.05 (ns)	−0.04 (ns)	−0.03 (ns)	−0.18 (ns)	−0.02 (ns)	0.19 (ns)	−0.13 (ns)	−0.05 (ns)
Treg activated	−0.11 (ns)	−0.07 (ns)	0.14 (ns)	−0.07 (ns)	−0.12 (ns)	−0.13 (ns)	0 (ns)	0.35 (*)	−0.16 (ns)	−0.07 (ns)
Th2 activated	−0.16 (ns)	−0.21 (ns)	0 (ns)	−0.22 (ns)	−0.12 (ns)	−0.11 (ns)	−0.30 (ns)	0.09 (ns)	−0.39 (**)	0.05 (ns)
Th17.Zhang	−0.22 (ns)	−0.23 (ns)	−0.09 (ns)	−0.26 (ns)	−0.2 (ns)	−0.23 (ns)	−0.04 (ns)	0.16 (ns)	−0.2 (ns)	−0.03 (ns)
ILC2_up_PMID26878113	−0.22 (ns)	−0.16 (ns)	0.11 (ns)	−0.24 (ns)	−0.24 (ns)	0.15 (ns)	0.18 (ns)	0.29 (ns)	0 (ns)	0.19 (ns)
ILC3_up_PMID26878113	−0.24 (ns)	−0.25 (ns)	0.21 (ns)	−0.28 (ns)	−0.14 (ns)	−0.14 (ns)	0 (ns)	0.51 (***)	−0.22 (ns)	−0.07 (ns)
Eosinophil	−0.26 (ns)	−0.18 (ns)	−0.08 (ns)	−0.24 (ns)	−0.24 (ns)	0.03 (ns)	0.13 (ns)	0.29 (ns)	−0.01 (ns)	0.29 (ns)

*Note:* Please see Table S2 for the details relating to asthma‐related gene signatures. The results are listed in descending order based on the correlation coefficient of the p53 signalling pathway score. Values within cells are rho (*p* value) calculated by Spearman test. *p*‐values were determined using Spearman's correlation analysis. To control for multiple hypothesis testing in this exploratory analysis, *p*‐values were adjusted using the Benjamini‐Hochberg method to control the false discovery rate (FDR). ***p < 0.001, **p < 0.01 and *p < 0.05 denote statistical significance based on FDR‐adjusted *p*‐values (*q*‐values).

Abbreviations: CD4, cluster of differentiation 4; GM‐CSF, granulocyte macrophage colony‐stimulating factor; HBEC, human bronchial epithelial cell; IFNG, interferon‐gamma; IL‐6, interleukin‐6; IL‐13, interleukin‐13; IL17α, interleukin‐17α; ILC, innate lymphoid cell; LPS, lipopolysaccharides; ns, not significant; OXPHOS, oxidative phosphorylation; SASP, senescence‐associated secretory phenotype; Th2, T helper 2 cell; Treg, regulatory T cell.

Conversely, the eosinophil signature ES did not show significant correlations with either the p53 or SASP scores. The Th2‐activated signature similarly demonstrated non‐significant correlations with the senescence signatures. In contrast, a significant positive correlation was observed between the SASP and the neutrophil signatures, particularly with the SASP functional cluster 1 and in both bronchial biopsies and nasal brushings (Table 4).

## 4 Discussion

This study leveraged the well‐characterised U‐BIOPRED cohort to demonstrate the significant enrichment of cellular senescence signatures, specifically the SASP and p53 signalling pathways, in the airways of patients with severe asthma. This enrichment was pronounced in bronchial biopsies and was associated with key clinical features of disease severity, including a history of frequent exacerbations, OCS use, fixed airflow obstruction and comorbid nasal polyps; the SASP enrichment was further validated in the independent NOVA cohort. Notably, nearly half of severe asthmatics fell into the co‐enriched (p53‐high/SASP‐high) quadrant. These findings provide translational evidence supporting a critical role for cellular senescence in driving the pathogenesis of severe asthma.

A notable finding of our study is the compartment‐specific enrichment of senescence signatures. The significant elevation of SASP and p53 scores according to asthma severity was observed exclusively in bronchial biopsies, which contain the full thickness of the airway wall, including the subepithelial and interstitial compartments, but not in superficial samples, such as bronchial or nasal brushings. This suggests that the burden of senescent cells is predominantly localised within the deeper structural tissues of the asthmatic airway, such as fibroblasts and smooth muscle cells, rather than solely in the superficial epithelium. Previous studies using in vitro models or isolated tissues have reported senescence markers (e.g., shortened telomeres, SA‐β‐gal, p21, p53) in asthmatic bronchial fibroblasts [15], epithelium [16] and smooth muscle cells [17, 29]. Our transcriptomic analysis of bronchial biopsies is consistent with prior observations and further demonstrates that activation of senescence pathways within the airway wall is a defining feature of severe disease. Notably, the absence of significant enrichment in bronchial brushings underscores the importance of sampling deeper airway tissues to accurately capture these pathological signatures.

The correlation of senescence signatures with clinical parameters of disease severity, particularly lower FEV1% and frequent exacerbations, supports the hypothesis of ‘accelerated airway ageing’ in severe asthma. The association with age of asthma onset, rather than chronological age, suggests that the chronic inflammatory and oxidative stress of the asthmatic airway accelerates biological ageing processes. This may also represent an effect of the altered host immune response to microbial presence observed in early‐onset allergic compared to late‐onset non‐allergic asthma [30]. The link with fixed airflow obstruction is also notable, proposing cellular senescence as a mechanistic driver of airway remodelling. This aligns with a previous finding in the BIOAIR cohort, where a severe asthma phenotype with persistent airflow limitation was associated with elevated serum levels of PAI‐1, a SASP factor and remodelling marker [31]. Furthermore, the positive relationship we observed between senescence scores and serum periostin, an extracellular matrix protein implicated in fibrosis and airway remodelling [32, 33], further substantiates the link between senescence and structural changes in the asthmatic airway.

Our study is the first to report a significant association between airway SASP signature expression and the presence of comorbid nasal polyps. In nasal brushing samples, SASP scores were significantly elevated in participants with nasal polyps compared to those without. Given that nasal polyps are typically adult‐onset [34, 35] and characterised by tissue remodelling, this finding suggests that cellular senescence may be a key pathogenic driver of nasal polyps, in addition to eosinophilic pathways. Intriguingly, SASP Cluster 2, which includes cell cycle regulators like CDKN1A (p21) and CDKN1B (p27), showed the strongest association with the presence of nasal polyps. Recent evidence of age‐related increases in collagen deposition and PAI‐1 in human nasal polyps further supports a senescence‐driven mechanism [36].

Our exploratory analysis using functional clusters and other gene signatures provides additional mechanistic insights. The strong correlation between the SASP Cluster 2 ES and the OXPHOS signature in nasal brushings (*r* = 0.78, *p* < 0.001) aligns with mitochondrial dysfunction and oxidative stress being potent drivers of senescence [37]. This suggests a metabolic basis for senescence in nasal polyps. Furthermore, in bronchial biopsies, we observed no significant correlation between senescence signatures and the eosinophil signature, whereas macrophage and CD4+ T‐cell activation signatures showed positive associations. We also found a significant correlation between the SASP signature, particularly Cluster 1 and the neutrophil signature in both airway compartments [38]. This suggests that cellular senescence is linked to a non‐T2 inflammatory component characterised by macrophage and neutrophil‐mediated inflammation and metabolic dysregulation (e.g., mitochondrial stress), rather than classic T2 inflammation. This distinction is clinically relevant, as it may explain poor responses to T2‐targeted therapies in some severe asthma patients.

Our findings are consistent with recent preclinical evidence demonstrating that airway smooth muscle cells from aged, allergen‐challenged mice exhibit senescence markers and contribute to airway remodelling and hyperresponsiveness [39]. Additionally, environmental exposure studies have reported altered p53 DNA methylation in moderate/severe asthma [19], supporting the involvement of the p53 pathway in asthma pathogenesis. It is important to note that T2 and non‐T2 pathways frequently coexist in severe asthma, and our findings suggest that cellular senescence contributes primarily to the non‐T2 component of the inflammatory milieu rather than defining a mutually exclusive endotype.

Although p53 and SASP capture distinct biological programmes of cellular senescence, most patients with severe asthma in our cohort fell into the dual‐high quadrant. A small subset of asthma patients showed a discordant pattern (only one pathway enriched) and appeared to represent an intermediate clinical phenotype, suggesting additive effects of the two pathways; however, the limited sample size precluded firm conclusions.

The major strengths of our study include the use of well‐characterised clinical cohorts with comprehensive clinical data, the analysis of multiple airway compartments and validation in an independent cohort. This study also has several limitations. First, the cross‐sectional design precludes the determination of causal relationships. Second, among the 107 subjects with available bronchial biopsy data, only 38 underwent longitudinal follow‐up, limiting the ability to assess prognosis and clinical course. Third, while statistically significant, some correlations between senescence scores and clinical parameters were weak. However, the associations with objective measures such as FEV1% and periostin were stronger. Fourth, nasal polyps were defined by physician diagnosis history, which in the majority of cases included otolaryngologist confirmation or a history of polypectomy, though systematic verification was not performed at study entry; in addition, we analysed nasal brushings rather than polyp tissue. Fifth, the p53 signalling pathway did not show a statistically significant association with asthma severity in the NOVA cohort. This may be attributable to the relatively small sample size. However, the direction of the effect was consistent with U‐BIOPRED (SA > MMA > HV), with a Cohen's *d* of 0.40 for SA versus HV, suggesting that the smaller sample size may have limited statistical power. Sixth, the study populations were predominantly Caucasian, limiting generalisability to other racial groups. Seventh, although we found no significant associations between chronological age and SASP/p53 scores, the long‐term effect of age on airway senescence warrants longitudinal investigation. Finally, we relied on transcriptomic signatures and did not directly quantify senescent cells or identify specific cell types in situ. Therefore, our findings should be interpreted as evidence of enrichment of senescence‐associated transcriptional programmes, rather than direct evidence of the senescent phenotype.

An important consideration is the potential confounding effect of OCS use. Glucocorticoids have been shown to induce cellular senescence via the p53/p21 pathway in primary human tenocytes [40], and a similar mechanism may contribute to p53 pathway activation in the airways of OCS‐treated severe asthma patients. Further studies are warranted to determine direct effects of corticosteroids on airway senescence mechanisms.

The U‐BIOPRED samples were collected between 2011 and 2014, prior to the widespread availability of biologic therapies targeting T2 inflammation. While this limits direct generalisability to patients currently on biologic therapy, it provides an assessment of senescence pathways unconfounded by biologic‐induced immune modulation. Future studies examining the effect of biologic therapies on senescence pathway expression would be of considerable interest.

In summary, our findings provide a clearer framework for interpreting the role of cellular senescence in severe asthma. First, senescence‐related pathways (SASP and p53) are enriched predominantly in bronchial biopsies, indicating that the deeper airway wall is the principal site of senescence‐associated changes. Second, higher senescence signature expression is consistently associated with key features of severe disease, including fixed airflow obstruction, frequent exacerbations and comorbid nasal polyps. Third, senescence signatures are linked to macrophage‐ and neutrophil‐associated, non‐T2 inflammatory pathways rather than eosinophilic/T2 inflammation, suggesting a distinct biological component within severe asthma.

Taken together, these findings suggest that patients with more pronounced senescence signatures represent a subgroup characterised by structural airway changes and non‐T2 inflammation, consistent with the concept of accelerated airway ageing. This framework may help to better define heterogeneity within severe asthma and guide future studies targeting senescence‐related pathways.

Further validation in independent cohorts and functional studies is required before these findings can be translated into clinical application. Whether targeting senescent cells or their secretory pathways may benefit patients with severe, treatment‐refractory asthma warrants further investigation, particularly given the emerging evidence for senolytic agents in other age‐related chronic diseases [41, 42, 43, 44].

## Author Contributions

W.‐J.S., N.Z.K. and A.V. analysed the data. Y.G. was responsible for the U‐BIOPRED dataset. S.S.‐O., J.L.S., P.A.W. and K.J.B. were responsible for the NOVA dataset. W.‐J.S., N.Z.K., I.M.A. and K.F.C. conceived the idea with contributions from S.‐E.D. and R.D. W.‐J.S., N.Z.K., I.M.A. and K.F.C. wrote the manuscript. All authors contributed to the revision and finalisation of the manuscript, and read and approved the final manuscript.

## Funding

U‐BIOPRED was supported by an Innovative Medicines Initiative Joint Undertaking (Grant 115010), resources from the European Union's Seventh Framework Programme (Grant FP7/2007–2013) and EFPIA companies' in‐kind contribution (www.imi.europa.eu). NOVA received funding from the John Hunter Hospital Charitable Trust. K.F.C. and I.M.A. are funded by UK Research and Innovation (UKRI). K.F.C. is an Emeritus Senior Investigator of the UK National Institute for Health Research (NIHR).

## Ethics Statement

All participants provided signed informed consent to participate in the study. The study was approved by the local Ethics Committees of participating centres.

## Conflicts of Interest

Dr. Song would like to declare he holds grants from Merck Sharp & Dohme Corp. and AstraZeneca, and receives financial enumeration from Merck, AstraZeneca, Shionogi and GSK (consultation fees) and from Merck, AstraZeneca, GSK, Sanofi, Novartis and Immunotek/ThermoFisher (lecture fees). Dr. Dahlén would like to declare support from IMI Grant (EU and EFPIA), Swedish Heart and Lung Foundation and ALF Clinical Research Stockholm County Council. Dr. Dahlén holds grants from AstraZeneca, Cayman Chemical and Sanofi and receives consulting fees from AstraZeneca, Cayman Chemical, GSK, Sanofi and Regeneron, outside the submitted work. He is an advisory group board member of the Swedish Asthma and Allergy Patient Association. Dr. Djukanovic would like to declare that he has stock or stock options at Synairgen and receives consulting fees from Synairgen and lecturing fees at symposia organised by Regeneron, GSK and Kymab. He also contributes to the Synairgen Data Safety Monitoring Board. Dr. Adcock holds grants from GSK, Medical Research Council, EPSRC and Sanofi, and receives consulting fees from GSK, Sanofi, Chiesi, lecture honoraria from AstraZeneca, Sanofi and Eurodrug and payments for education events organised by Sunovion. Dr. Adcock also receives support from AstraZeneca to travel and attend meetings. Additionally, Dr. Adcock is a member of advisory board of Kinaset. Dr. Chung has received honoraria for participating in advisory board meetings of GSK, AZ, Roche, Novartis, Merck, BI, TEVA and Shionogi, contributing his expertise to matters relating to treatments for asthma, chronic obstructive pulmonary disease and chronic cough. Dr. Chung has also been renumerated for speaking engagements. Other authors have nothing to declare.

## Supporting information


**Data S1:** all70416‐sup‐0001‐Supinfo1.docx.

**Table S1:** Cellular senescence‐related signatures used for the gene set variation analyses.

**Table S2:** Gene sets representing specific mechanisms or pathways in asthma.

**Table S3:** Comparison of senescence enrichment scores between severe asthma patients on daily maintenance OCS and those not on OCS.

**Table S4:** Multivariate linear regression between clinical parameters and senescence enrichment scores in bronchial biopsies of asthma patients (*n* = 81).

**Table S5:** Comparison of clinical and demographic characteristics according to the p53/SASP quadrant classification.

**Table S6:** Multivariate linear regression between clinical parameters and the SASP cluster gene set enrichment scores in (A) bronchial biopsies and (B) nasal brushing.

## Data Availability

The datasets generated and analysed during the current study are not publicly available as the data is managed by the U‐BIOPRED consortium. However, the study data is available from the corresponding author on reasonable request.

## Appendix

List of the U‐BIOPRED Study Group Members: Mahmoud. I. Abdel‐Aziz^1^, Ian M. Adcock^2^, Lars I. Andersson^3^, Charles Auffray^4^, Yusef E. Badi^2^, Per Bakke^5^, Aruna T. Bansal^6^, Frederic Baribaud^7^, Stewart A. Bates^8^, Elisabeth H. Bel^9^, Jeanette Bigler^10^, Bo Billing^3^, Hans Bisgaard^11^, Michael J. Boedigheimer^12^, Klaus Bønnelykke^11^, Joost Brandsma^13^, Paul Brinkman^9^, Enrica Bucchioni^14^, Dominic Burg^15^, Andrew Bush^16^, Massimo Caruso^17^, Romanas Chaleckis^18^, Pascal Chanez^19^, Kian Fan Chung^2^, T. Checa^18^, Chris H. Compton^20^, Julie Corfield^21^, Danen Cunoosamy^22^, Arnaldo D'Amico^23^, Barbro Dahlén^24^, Sven‐Erik Dahlén^25^, Bertrand De Meulder^26^, Ratko Djukanovic^27^, Veit J. Erpenbeck^28^, Damijan Erzen^29^, K. Fichtner^29^, Louise J. Fleming^16^, Elena Formaggio^30^, Stephen J. Fowler^31^, Urs Frey^32^, Martina Gahlemann^33^, Thomas Geiser^34^, Victoria Goss^35^, Yike Guo^36^, Simone Hashimoto^9^, John Haughney^37^, Gunilla Hedlin^38^, Pieter‐Paul W. Hekking^9^, Timothy Higenbottam^39^, Jens M. Hohlfeld^40^, Cecile Holweg^41^, Ildiko Horvath^42^, Peter Howarth^43^, Anna J. James^25^, Richard G. Knowles^44^, Johan Kolmert^18^, Jon Konradsen^38^, Norbert Krug^45^, Nikos Lazarinis^3^, C.‐X. Li^46^, Matthew J. Loza^7^, Rene Lutter^9^, Alexander Manta^47^, Sarah Masefield^48^, Anke H. Maitland‐van der Zee^1^, John G. Matthews^41^, Alexander Mazein^4^, Roelinde J. M. Middelveld^25^, Montserrat Miralpeix^49^, Paolo Montuschi^50^, Jacek Musial^51^, Sharon Mumby^2^, David Myles^8^, Björn Nordlund^38^, Ioannis Pandis^36^, Stelios Pavlidis^2^, Anthony Postle^13^, P. Powel^48^, G. Praticò^52^, M. Puig Valls^53^, N. Rao^7^, Stacey Reinke^18^, John Riley^8^, Amanda Roberts^54^, Graham Roberts^43^, Anthony Rowe^7^, Thomas Sandström^55^, Jim P. R. Schofield^15^, Wolfgang Seibold^29^, Dominic E. Shaw^56^, R. Sigmund^29^, Florian Singer^57^, Paul J. Skipp^15^, M. Smicker^22^, Ana R. Sousa^8^, Maria Sparreman‐Mikus^58^, Peter J. Sterk^9^, Marika Ström^46^, Kai Sun^36^, Bob Thornton^59^, Mohib Uddin^60^, Ali Versi^3^, Jorgen Vestbo^31^, Nadja H. Vissing^11^, Scott S. Wagers^61^, Åsa Wheelock^62^, Craig E. Wheelock^25^, Susan J. Wilson^63^, Valentyna Yasinska^58^, Nazanin Zounemat Kermani^36^

^1^Department of Respiratory Medicine, Amsterdam UMC, University of Amsterdam, Amsterdam, the Netherlands

^2^National Heart and Lung Institute, Imperial College, London, UK

^3^Department of Respiratory Medicine, Karolinska University Hospital, Stockholm, Sweden

^4^European Institute for Systems Biology and Medicine, CNRS‐ENS‐UCBL‐INSERM, Lyon, France

^5^Department of Clinical Science, University of Bergen, Bergen, Norway

^6^Acclarogen, St John's Innovation Centre, Cambridge, UK

^7^Janssen Research and Development, High Wycombe, Buckinghamshire, UK

^8^Respiratory Therapeutic Unit, GlaxoSmithKline, London, UK

^9^Academic Medical Centre, University of Amsterdam, Amsterdam, the Netherlands

^10^Previously Amgen, Thousand Oaks, California, USA

^11^Copenhagen Prospective Studies on Asthma in Childhood, Herlev and Gentofte Hospital, University of Copenhagen, Copenhagen, Denmark

^12^Amgen, Thousand Oaks, California, USA

^13^University of Southampton, Southampton, UK

^14^Chiesi Pharmaceuticals SPA, Parma, Italy

^15^Centre for Proteomic Research, Institute for Life Sciences, University of Southampton, Southampton, UK

^16^National Heart and Lung Institute, Imperial College, London, UK, and Royal Brompton and Harefield NHS Trust, London, UK

^17^Department of Clinical and Experimental Medicine, University of Catania, Catania, Italy

^18^Institute of Environmental Medicine, Centre for Allergy Research, Karolinska Institutet, Stockholm, Sweden

^19^Assistance publique des Hôpitaux de Marseille—Clinique des bronches, allergies et sommeil, Aix Marseille Université, Marseille, France

^20^Respiratory Therapeutic Unit, GlaxoSmithKline, London, UK

^21^Areteva R&D, Nottingham, UK

^22^Sanofi, Cambridge, Massachusetts

^23^University of Rome Tor Vergata, Rome, Italy

^24^Karolinska University Hospital & Centre for Allergy Research, Karolinska Institutet, Stockholm, Sweden

^25^Centre for Allergy Research, Karolinska Institutet, Stockholm, Sweden

^26^European Institute for Systems Biology and Medicine, CNRS‐ENS‐UCBL‐INSERM, Lyon, France

^27^NIHR Southampton Respiratory Biomedical Research Unit and Clinical and Experimental Sciences, Southampton, UK

^28^Translational Medicine, Respiratory Profiling, Novartis Institutes for Biomedical Research, Basel, Switzerland

^29^Boehringer Ingelheim PharmaGmbH& Co. KG, Biberach, Germany

^30^Previously CROM‐SOURCE, Verona, Italy

^31^Centre for Respiratory Medicine and Allergy, Institute of Inflammation and Repair, University of Manchester and University Hospital of South Manchester, Manchester Academic Health Sciences Centre, Manchester, UK

^32^University Children's Hospital, Basel, Switzerland

^33^Boehringer Ingelheim (Schweiz), Basel, Switzerland

^34^Department of Respiratory Medicine, University Hospital Bern, Bern, Switzerland

^35^NIHR Respiratory Biomedical Research Unit, University Hospital Southampton NHS Foundation Trust, Integrative Physiology and Critical Illness Group, Clinical and Experimental Sciences, Sir Henry Wellcome Laboratories, Faculty of Medicine, University of Southampton, Southampton, UK

^36^Data Science Institute, Imperial College, London, UK

^37^International Primary Care Respiratory Group, Aberdeen, UK

^38^Department of Women's and Children's Health & Centre for Allergy Research, Karolinska Institutet, Stockholm, Sweden

^39^Allergy Therapeutics, West Sussex, UK

^40^Fraunhofer Institute for Toxicology and Experimental Medicine, Hannover, Germany

^41^Respiratory and Allergy Diseases, Genentech, San Francisco, California, USA

^42^Semmelweis University, Budapest, Hungary

^43^NIHR Southampton Respiratory Biomedical Research Unit, Clinical and Experimental Sciences and Human Development and Health, Southampton, UK

^44^Arachos Pharma, Stevenge, UK

^45^Fraunhofer Institute for Toxicology and Experimental Medicine, Hannover, Germany

^46^Department of Medicine Solna, Karolinska Institutet, Stockholm, Sweden

^47^Roche Diagnostics GmbH, Mannheim, Germany

^48^European Lung Foundation, Sheffield, UK

^49^Almirall, Barcelona, Spain

^50^Università Cattolica del Sacro Cuore, Milan, Italy

^51^Department of Medicine, Jagiellonian University Medical College, Krakow, Poland

^52^CROMSOURCE, Verona, Italy

^53^CROMSOURCE, Barcelona, Spain

^54^Asthma UK, London, UK

^55^Department of Public Health and Clinical Medicine, Umeå University, Umeå, Sweden

^56^Respiratory Research Unit, University of Nottingham, Nottingham, UK

^57^University Children's Hospital, Zurich, Switzerland

^58^Department of Medicine Huddinge, Karolinska Institutet, and Department of Respiratory Medicine, Karolinska University Hospital, Stockholm, Sweden

^59^MSD, Rathdrum, Ireland

^60^AstraZeneca BioPharmaceuticals R&D, Gothenburg, Sweden

^61^BioSci Consulting, Maasmechelen, Belgium

^62^Respiratory Medicine Unit, Department of Medicine Solna and Center for Molecular Medicine, Karolinska Institutet, Stockholm, Sweden and Department of Respiratory Medicine and Allergy, Karolinska University Hospital Solna, Stockholm, Sweden

^63^Histochemistry Research Unit, Faculty of Medicine, University of Southampton, Southampton, UK
