# Supplementary material for: Clinical Features of Cellular Senescence Pathways in Severe Asthma
Source: Allergy. 2026 Jun 18;81(9):3273–85. doi: 10.1111/all.70416 (PMC13569611; doi:10.1111/all.70416)
Supplement: Supplementary file 1 [file ALL-81-3273-s001.docx]

**Table S6. Multivariate linear regression between clinical parameters and the SASP cluster gene set enrichment scores in (A) bronchial biopsies and (B) nasal brushing**

| **(A) Bronchial biopsy** | **SASP cluster 1 ES** | | **SASP cluster 2 ES** | | **SASP cluster 3 ES** | | |
| --- | --- | --- | --- | --- | --- | --- | --- |
| Parameters | Regress. Coeff. (95% CI)† | *P* value | Regress. Coeff. (95% CI)† | *P* value | Regress. Coeff. (95% CI)† | *P* value | |
| Asthma onset age (years) | 0.002 (-0.003 to 0.005) | 0.273 | 0.003 (-0.001 to 0.008) | 0.097 | 0.005 (0.000 to 0.009) | 0.042 | |
| Exacerbations in the previous year (number)* | 0.040 (0.007 to 0.072) | 0.019 | 0.052 (0.010 to 0.010) | 0.017 | 0.070 (0.021 to 0.120) | 0.006 | |
| Maintenance OCS use (vs. no) | 0.189 (0.095 to 0.283) | <0.001 | 0.185 (0.047 to 0.323) | 0.009 | 0.173 (0.012 to 0.334) | 0.036 | |
| Nasal polyps (vs. no) | 0.163 (0.054 to 0.271) | 0.004 | 0.233 (0.094 to 0.371) | 0.001 | 0.134 (-0.034 to 0.303) | 0.117 | |
| Post-BD FEV1% pred (%) | -0.011 (-0.208 to 0.186) | 0.910 | -0.401 (-0.661 to 0.141) | 0.003 | -0.297 (-0.606 to 0.012) | 0.059 | |
| Serum periostin (ng/ml)* | 0.252 (0.053 to 0.452) | 0.014 | 0.229 (-0.027 to 0.485) | 0.079 | 0.315 (0.006 to 0.623) | 0.046 | |
| **(B) Nasal brushing** | **SASP cluster 1 ES** | | **SASP cluster 2 ES** | | **SASP cluster 3 ES** |  |  |
| Parameters | Regress. Coeff. (95% CI)†† | *P* value | Regress. Coeff. (95% CI)†† | *P* value | Regress. Coeff. (95% CI)†† | *P* value | |
| Nasal polyps (vs. no) | 0.138 (-0.017 to 0.294) | 0.081 | 0.202 (0.042 to 0.362) | 0.014 | 0.223 (-0.002 to 0.447) | 0.052 | |

Values indicate regression coefficients (95% confidence intervals).

*Variable log-transformed to normalize distribution.

†Adjusted for age, sex, smoking, and body mass index.

††Adjusted for age, sex, smoking, asthma, and nasal polyps.

Abbreviations: 95% CI, 95% confidence interval; ES, enrichment score; FEV1, forced expiratory volume in 1 second; OCS, oral corticosteroids; post-BD, post-bronchodilator; SASP, senescence-associated secretory phenotype
