# Supplementary material for: Clinical Features of Cellular Senescence Pathways in Severe Asthma
Source: Allergy. 2026 Jun 18;81(9):3273–85. doi: 10.1111/all.70416 (PMC13569611; doi:10.1111/all.70416)
Supplement: Supplementary file 2 [file ALL-81-3273-s002.docx]

**Table S3. Comparison of senescence enrichment scores between severe asthma patients on daily maintenance OCS and those not on OCS**

| **Parameter** | **Daily OCS (n=19)** | **No daily OCS (n=34)** | ***P* value** |
| --- | --- | --- | --- |
| Age (years) | 57.0 (41.5, 62.5) | 49.0 (39.5, 56.8) | 0.028 |
| BMI (kg/m²) | 30.5 (25.6, 35.4) | 28.4 (24.6, 32.1) | 0.239 |
| Post-BD FEV1% | 79.8 (73.4, 87.6) | 79.2 (64.0, 95.1) | 0.948 |
| Exacerbations/year | 9.0 (2.5, 11.0) | 9.0 (2.0, 11.0) | 0.606 |
| p53 ES | 0.133 (-0.024, 0.324) | -0.041 (-0.165, 0.185) | 0.068 |
| SASP ES | 0.117 (-0.026, 0.294) | 0.023 (-0.136, 0.130) | 0.073 |

OCS, oral corticosteroids, BMI, body mass index; BD, bronchodilator; FEV1, forced expiratory volume in 1 second; SASP, senescence-associated secretory phenotype; ES, enrichment score
