# Supplementary material for: Clinical Features of Cellular Senescence Pathways in Severe Asthma
Source: Allergy. 2026 Jun 18;81(9):3273–85. doi: 10.1111/all.70416 (PMC13569611; doi:10.1111/all.70416)
Supplement: Supplementary file 3 [file ALL-81-3273-s003.docx]

**Online Supplementary Materials**

***Results***

*Functional cluster exploration of the SASP gene set*

To address the large number of genes and the functional heterogeneity within the SASP gene set, we used protein-protein interaction data from STRINGDB (https://string-db.org) and applied K-means clustering to organize the proteins into three highly interconnected functional clusters (Figure 3A).

Cluster 1 was enriched for REACTOME pathways related to oxidative stress-induced senescence (p = 7.33 × 10⁻¹³), RSK activation (p = 1.69 × 10⁻¹⁰), and IL-6 signaling (p = 0.002). Genes in this cluster have been associated with omalizumab treatment responses in airway smooth muscle cells^1^, airway inflammation^1^, atopic asthma^2^, PI3K inhibitor treatment^3^, functional translation of IL1RL1 locus polymorphisms in lung tissue and asthmatic airway epithelium^4^, mechanism of TH2/TH17-predominant and neutrophilic TH2/TH17-low subtypes of asthma.^5^

Cluster 2 included histone (HIST) proteins, while CDKN1B and CCNA2 are reported to regulate the activation and proliferation of T lymphocytes in asthma.^6^ CDKN1B, CDKN1A and H2AFX are reported to have a role in lung injury and repair.^7^ Other genes in this cluster have been reported in the context of lung cancer.^8-12^

Cluster 3 includes HIST (1/2/3/4) H family and H (2/3) genes. Cluster 3 genes are reported by studies of steroid glucocorticoid-resistant asthma^13-15^, environmental effects on immune response^16^ and inflammation and remodeling.^17-20^

**References**

1.	Roth M, Tamm M. The effects of omalizumab on IgE-induced cytokine synthesis by asthmatic airway smooth muscle cells. *Ann Allergy Asthma Immunol.* 2010;104:152-160.

2.	Xu Z, Ye Y, Huang G, et al. EphA2 recognizes Dermatophagoidespteronyssinus to mediate airway inflammation in asthma. *Int Immunopharmacol.* 2022;111:109106.

3.	Bi J, Min Z, Yuan H, et al. PI3K inhibitor treatment ameliorates the glucocorticoid insensitivity of PBMCs in severe asthma. *Clin Transl Med.* 2020;9:22.

4.	Portelli MA, Dijk FN, Ketelaar ME, et al. Phenotypic and functional translation of IL1RL1 locus polymorphisms in lung tissue and asthmatic airway epithelium. *JCI Insight.* 2020;5.

5.	Liu W, Liu S, Verma M, et al. Mechanism of TH2/TH17-predominant and neutrophilic TH2/TH17-low subtypes of asthma. *Journal of Allergy and Clinical Immunology.* 2017;139:1548-1558. e1544.

6.	Zhang W, Nie Y, Chong L, et al. PI3K and Notch signal pathways coordinately regulate the activation and proliferation of T lymphocytes in asthma. *Life sciences.* 2013;92:890-895.

7.	Habiel DM, Camelo A, Espindola M, et al. Divergent roles for Clusterin in Lung Injury and Repair. *Sci Rep.* 2017;7:15444.

8.	Zhou J, Zhang S, Fu G, et al. Overexpression of APC11 predicts worse survival in lung adenocarcinoma. *Onco Targets Ther.* 2018;11:7125-7132.

9.	Wu C-C, Huang K-F, Yang T-Y, et al. The Topoisomerase 1 inhibitor Austrobailignan-1 isolated from Koelreuteria Henryi induces a G2/M-phase arrest and cell death independently of p53 in non-small cell lung cancer cells. *PLoS One.* 2015;10:e0132052.

10.	Sugiyama T, Frazier DP, Taneja P, Morgan RL, Willingham MC, Inoue K. Role of DMP1 and its future in lung cancer diagnostics. *Expert Rev Mol Diagn.* 2008;8:435-447.

11.	Pai J-T, Hsu M-W, Leu Y-L, Chang K-T, Weng M-S. Induction of G2/M Cell Cycle Arrest via p38/p21Waf1/Cip1-Dependent Signaling Pathway Activation by Bavachinin in Non-Small-Cell Lung Cancer Cells. *Molecules.* 2021;26:5161.

12.	Ge H, Ni S, Wang X, et al. Dexamethasone reduces sensitivity to cisplatin by blunting p53-dependent cellular senescence in non-small cell lung cancer. *PLoS One.* 2012;7:e51821.

13.	Corrigan CJ, Loke TK. Clinical and molecular aspects of glucocorticoid resistant asthma. *Ther Clin Risk Manag.* 2007;3:771-787.

14.	Chang PJ, Michaeloudes C, Zhu J, et al. Impaired nuclear translocation of the glucocorticoid receptor in corticosteroid-insensitive airway smooth muscle in severe asthma. *Am J Respir Crit Care Med.* 2015;191:54-62.

15.	Zhang Y, Leung DY, Goleva E. Anti-inflammatory and corticosteroid-enhancing actions of vitamin D in monocytes of patients with steroid-resistant and those with steroid-sensitive asthma. *Journal of Allergy and Clinical Immunology.* 2014;133:1744-1752. e1741.

16.	Miller RL, Peden DB. Environmental effects on immune responses in patients with atopy and asthma. *J Allergy Clin Immunol.* 2014;134:1001-1008.

17.	Calvo FQ, Fillet M, de Seny D, et al. Biomarker discovery in asthma-related inflammation and remodeling. *Proteomics.* 2009;9:2163-2170.

18.	Clifford RL, John AE, Brightling CE, Knox AJ. Abnormal histone methylation is responsible for increased vascular endothelial growth factor 165a secretion from airway smooth muscle cells in asthma. *The Journal of Immunology.* 2012;189:819-831.

19.	Herbert C, Shadie AM, Bunting MM, et al. Anti-inflammatory and anti-remodelling effects of ISU201, a modified form of the extracellular domain of human BST2, in experimental models of asthma: association with inhibition of histone acetylation. *PLoS One.* 2014;9:e90436.

20.	Clifford RL, Patel JK, John AE, et al. CXCL8 histone H3 acetylation is dysfunctional in airway smooth muscle in asthma: regulation by BET. *American Journal of Physiology-Lung Cellular and Molecular Physiology.* 2015;308:L962-L972.
