# Supplementary material for: Clinical Features of Cellular Senescence Pathways in Severe Asthma
Source: Allergy. 2026 Jun 18;81(9):3273–85. doi: 10.1111/all.70416 (PMC13569611; doi:10.1111/all.70416)
Supplement: Supplementary file 4 [file ALL-81-3273-s004.docx]

**Table S2. Gene sets representing specific mechanisms or pathways in asthma**

| **Signature names** | **Genes** | **Ref** |
| --- | --- | --- |
| IL-13 stimulated HBECS | CST1, CCL26, PRB2, PRB1, PRB3, POSTN, PRB4, ITLN1, ALOX15, SH2D1B, CA2, NOS2, FCGBP, FOXA3, SPDEF, CAPN14, DUOXA2, CLDN5, PADI3, TSPAN8, ALPL, KCNJ16, FETUB, B3GNT6, CDH26, LRRC31, MUC13, VSIG2, CSTA, FAM3B, SLC9B2, NTRK1, KLF4, HPDL, SOCS1, TRNP1, HS3ST1, VWF, DUOX2, CISH, ATP13A5, ZNF808, RNASE4, CCBL1, SDCBP2, TMPRSS2, HYAL1, CCDC109B, FAM83D, TRAK1, TPK1, SLC7A1, CYP2C18, CDC42EP5, KCNS3, ADRA2A, MRAP2, SLC2A10, PPARG, FAM26E, ADCY4, WNT3, SLCO4A1, ALDH1A2, C10orf99, WDFY2 | ^1^ |
| ILC1 cells | SIT1, CD3D, CD3G, CD4, CD6, TRAV13-1, CD5, CD27, C14orf64, COTL1, CD8A, IKZF3, LITAF, CCR7, TRAV8-2, TRAV4, SYNE2..1, MAL, GZMM, GZMK, TC2N, GZMA, SH2D1A, IFNG-AS1, PASK, TRBV5-1, ADTRP, TRAV9-2, CACNA1I, CCL5, ACTN1, CXCR3, BCL11B, PYHIN1, CH25H, LBH, FBLN7, LINC00402, TRAV2, TRBV2, IGFBP3, ANK3 ,IL6R, LDLRAP1, ACSL6, TRAV41, MIAT, TRBV20-1, LAG3, IFNG, TRAV26-2, GABBR1, TSHZ2, SLC25A4, AP000569.8..1, RASGRF2, TRAV8-4, RP11-664D1.1, TNFRSF10D, PLEKHB1, TRAV12-2 | ^2^ |
| Inflammasome | IL1B, NLRP3, CASP1, CASP4, CASP5 | ^3^ |
| OXPHOS | ND1, ND2, ND3, ND4, ND4L, ND5, ND6, NDUFS1, NDUFS2, NDUFS3, NDUFS4, NDUFS5, NDUFS6, NDUFS7, NDUFS8, NDUFV1, NDUFV2, NDUFV3, NDUFA1, NDUFA2, NDUFA3, NDUFA4, NDUFA4L2, NDUFA5, NDUFA6, NDUFA7, NDUFA8, NDUFA9, NDUFA10, NDUFAB1, NDUFA11, NDUFA12, NDUFA13, NDUFB1, NDUFB2, NDUFB3, NDUFB4, NDUFB5, NDUFB6, NDUFB7, NDUFB8, NDUFB9, NDUFB10, NDUFB11, NDUFC1, NDUFC2, NDUFC2-KCTD14, SDHA, SDHB, SDHC, SDHD, UQCRFS1, CYTB, CYC1, UQCRC1, UQCRC2, UQCRH, UQCRHL, UQCRB, UQCRQ, UQCR10, UQCR11, COX10, COX3, COX1, COX2, COX4I2, COX4I1, COX5A, COX5B, COX6A1, COX6A2, COX6B1, COX6B2, COX6C, COX7A1, COX7A2, COX7A2L, COX7B, COX7B2, COX7C, COX8C, COX8A, COX11, COX15, COX17, ATP5A1, ATP5B, ATP5C1, ATP5D, ATP5E, ATP5O, ATP6, ATP5F1, ATP5G1, ATP5G2, ATP5G3, ATP5H, ATP5I, ATP5J2, ATP5L, ATP5J, ATP8, ATP6V1A, ATP6V1B1, ATP6V1B2, ATP6V1C2, ATP6V1C1, ATP6V1D, ATP6V1E2, ATP6V1E1, ATP6V1F, ATP6V1G1, ATP6V1G3, ATP6V1G2, ATP6V1H, TCIRG1, ATP6V0A2, ATP6V0A4, ATP6V0A1, ATP6V0C, ATP6V0B, ATP6V0D1, ATP6V0D2, ATP6V0E1, ATP6V0E2, ATP6AP1, ATP4A, ATP4B, ATP12A, PPA2, PPA1, LHPP | ^4^ |
| Neutrophil | ABTB1, AMPD2, C5orf6, CCR3, CDA, CKLFSF2, CLC, CREB5, CTBS, DcR1, EST, FCGR2B, FCGR3B, FLJ10298, FPRL1, FRAT2, GPR27, GPR43, HSPA6, IL8RA, IL8RB, KIAA0779, KIAA1126, KRT23, LENG4, LENG5, MAD, MGC10500, MGC14126, MGC16353, MPPE1, MSCP, NCF4, NRBF2, PHC2, PROK2, RALB, RNF141, SEC14L1, SEPX1, STX3A, TM4-B, VMP1, VNN2, XPO6 | ^5^ |
| LPS-stimulated macrophage | MLLT4, ELL2, NMT2, SLC43A3, IL4I1, MCOLN2, MAOA, ZFP36L1, APOBEC3A, TXN, FTH1, CCL7, ACSL1, CCL23, VWA8, RBM47, RPLP1, HSP90B3P, RHBDF2, RPN2, HNRNPC, HMGN2P46, SDF4,FABP5, ANXA2P1, TNIP1, TNIP3, LILRB1, TIMP1, PSTPIP2, MARCKSL1, ADAMDEC1, SLC3A2, SRC, NMI, CYP27B1, KYNU, WSB2, SLC41A2, IL3RA, S100A12, VRK2, YWHAG, CXCL5, ATP2C1, NFS1, BID, MTF1, PPBP, PPP2CB, PDE4DIP, CCL5, SLC2A6, CXCL6, CD40, FABP5P3, IFIH1, LAD1, SDC2, GPR84, C1orf122, LILRA3, CFLAR, CAPZA1, LGALS3, GCH1, EIF1AX, P2RX4, NFE2L3, SLBP, CLIC4, MMP12, ACP2, MYC, ARL6IP1, ATP11B, ZSWIM4, VPS37A, SRGN, FTH1P3, TDP2, OAS3, C6orf211, SMAD7, SMARCA5, HSPA13, GPBP1, PARP10, BOLA2, NUP62, IL7R, G3BP1, LARP4, LILRB4, ILK, SLC16A3, GRINA, C1QTNF1, SOD2, FAM129B,PS29, TPI1, LOC440704, HSPB1, MT1H, EXOG, CD68, PTMA, MT1X, KIAA1147, PDIA3P1, MT1IP, MT1E, GYPC, TPT1, SLC29A1 | ^6^ |
| IFNG-stimulated macrophage | SELM, EHD1, IL10RA, NAMPT, MCTP1, C15orf48, SLC7A11, SIGLEC10, CDK5RAP2, EREG, TERF2IP, IL1A, ISG20, MYH11, MCOLN2, CLCF1, DDIT4, ANKRD12, AARS, SQSTM1, FLNB, NUB1, DCUN1D3, CXCL8, RILPL2, DRAM1, ATOX1, PTGS2, MAP3K4, UPB1, ADARB1, CREG1, PTPN1, RNF19B, IER3, NKG7, FAM60A, IL2RA, SLC7A1, MT1H, SAMSN1, ASCC1, FYN, MT2A, ETS1, PPP3CC, MT1A, LAT, RHBDF2, G0S2, CEP135, PANX2, C5orf15, HMGN2P46, ORM1, TRAF1, C17orf58, MB21D2, LPAR1, TRAF3IP2, TNFAIP3, SEMA4A, ARHGEF2, LMO4, TNIP1, TBC1D9, ACSL5, CYP2S1, BTG3, TNIP3, EXOG, NLRP3, SEMA6B, PSTPIP2, PTGES, MARCKSL1, CD86, NFKB1, LSS, AQP9, TANK, CCR7, MT1G, LAMB3, SLC25A37, RUNX2, ADAMDEC1, NFKBIE, SYT11, PVRL2, MLLT6, MT1F, ETS2, SLC7A5, CA12, MRPS6, GK, SLC3A2, BHLHE41, WARS, TNFAIP2, CXCL16, PILRA, CCDC115, TMEM205, HIST2H2BE, SIK3, ITGB8, PPP1R18, PTPRE, NBN, HIVEP1, GRAMD1A, KYNU, SLC1A3, NFE2L1, PCNX, CD14, CYP27B1, APBB3, FPR2, CEP350, CTTN, IRAK2, DDX59, CCM2L, PIM2, TSPAN33, ACO1, TAP1, RNF144B, CXCL5, IKBKE, EMR3, APOL3, H1F0, NADK, ZMIZ2, NFS1, HLA-F, BATF3, HIF1A, MTF1, YARS, STOM, RASGRP1, HIST2H2AC, GJB2, SLAMF1, CLEC4E, CCL5, KIAA0226L, GNA15, CFB, AMPD3, PNKD, MT1X, SMOX, IL1B, BIRC3, MAP1LC3A, ZMYND15, SLC2A6, SPECC1L, OSM, PRKCH, C1S, LAD1, PDE4B, STAT4, GPR84, TNFAIP6, C1orf122, PLAGL2, SLC43A2, HELZ2, TSC22D1, SLC5A3, CXCL1, HEY1, ABHD17C, CRISPLD2, DNAJB6, PGD, NDP, MMP9, ASNS, IRF7, ACKR3, RCN1, SLC9A1, RFTN1, EBI3, TGFA, PPA1, ADORA2A, CLEC4D, MUCL1, GPR132, RELB, FAM20A, GCH1, CTSL, NINJ1, ST3GAL1, SMAP2, MYO1B, TYMP, TNFRSF4, NCF1C, GBP5, HBEGF, CYB561A3, MT1IP, ZSWIM4, IL36G, LAMP3, MT1E, ZHX2, PFKFB3, N4BP1, MXD1, GNA12, CD38, PARP10, DTX2, LILRB2, TMEM132A, EMR2, SPHK1, SIRPA, PLAUR, VEGFA, PTPRF, C1QTNF1, STEAP3, MPZL1, CD82, CLEC4A, DUSP10, GLIS3, SOD2, ISCU | ^6^ |
| ILC2_up PMID26878113 | HPGDS, KRT1, IL17RB, TNFRSF19, PTGDR2, HPGD, IL1RL1, PKIB, C10orf128, RP11345M22.1, FSTL4, GAP43, MBOAT2, KLRG1, CSGALNACT1, FCRL3, CLIC4, IL10RA, HLF, LGALS12, ZP1, CHDH, RP11440I14.2, A2M, BACE2, RP11345M22.2, P2RY1, FASLG, NRIP3, MSRB3, NTRK1, LINC00340, PZP, PPARG, TNFRSF9, UBXN10, A2MP1, IFIT3, UTS2, CALCRL, RAP1GAP2, GRK5 | ^2^ |
| Th17 Zhang | KLRB1, RORC, PLXND1, CTSH, ALOX5, PTPN13, IL4I1, C11orf75, NEFL, HLF, JAKMIP2, DSE, LIMS1, HLA-DRB1, LTK, HLA-DRB4, USP10, NR1D1, LCAT, SAMD3, HSPG2, PTGIR | ^7^ |
| ILC3_up PMID26878113 | FCER1G, SH2D1B, NCR2, NRP1, LINC00299, KIAA1324, LST1, AMICA1, IL23R, PCDH9, VWA5A, XCL1, SIGLEC7, PLCG2, KLRC1, IL4I1, SLC4A10, KIT, GSN, IL1R1, TOX2, CD300LF, TYROBP, PTGDR, KRT81, XCL2, LTA4H, SPINK2, STAC, MYO7A, OTUD5, KLRF2, LIF, AFF3, ATP8B4, ENPP1, IL2RB, TMIGD2, PRR5, ELOVL6, AC092580.4, TNFSF13B, AFAP1L1, TGM2, B3GNT7, COL23A1, ENG, LDLRAD3, FGR, TNFRSF18, HIP1, APOL4, RP11-845M18.6, ITM2C, PLCH1, LPAR1, RHOC, NSMCE1, SCN1B, ID2, SLC43A2, ABCB5, ADAM28, CAT, RP11-563D10.1, TNFSF11, STARD3NL, TNS3, COL4A4, RNF152, CA2, TOX, FES, KIAA1217, B4GALNT1, VAV3, GPR82, DOCK5, CD33, S100A13, SOST, TLE1, PECAM1, BCAS1, RORC, LRRN3, TLE3, NCR1, SYK, HDAC9, CRTAM, DOK3, TNFSF4, CD300C, RP11330A16.1, FSD1, GNLY, CLNK, CXXC5, HPN, GOLIM4, AP000476.1, SPRY1, FAM179A, MPG, ABHD15, SPRED2, TRAJ45, GPR68, PRAM1, PDE6G, MATN2, AE000661.37, DOK7, ARMC9, HOXA10, SERPINA11, PDZK1, ENTPD1, LINGO4, TTN, CCL20, CD81, HOXA5, AC017104.6, SERP2, TRGJP1, SNCA, NEK10, TCF4, RP11-428G5.5, RGS9, KIAA0087, IRF4, DACH1, NCAM1, IKZF2, CDR2L, FCRL4, PVRL2, LTBR, NLRP7, JAG2, TRDJ2, RP1191K8.1, S100B, LDLRAD4..4, HOXA7..1, CHKA, EFCAB4A, SUOX, KCTD11, RP11-31E23.1..1, RP11-98D18.9..2, LIMK1, JUP, RP11-15B24.5, KLRK1..1, KIFC3, RP11-98G13.1, IGFBP4, BANK1, OPCML..1, DTWD2, REEP1, MEF2C, ZFYVE9, MECOM, CARD9, GPRC5C, MUC2, INPP1, MPV17L, MYB, SLC2A10, SLCO2A1, GRAMD1B, CARD6, ARSJ, CHMP4C, SNAP91, KCNQ5, TSPAN13, NLRP2, LYN, RP11256L6.2, CXCL16, CCDC102B, GSAS1, C19orf35, MACC1, SORT1, GPR133, CCDC171, ORAI3, SERPINH1..1, NPTXR, PTPRD, DERL3, PPP1R9A, B3GALT5, P4HA2, TRAJ44, MRC2, SLFN12, PLEKHN1, ACP6, TIE1, TMED8, UNC93B1, RP11279F6.3, RP11510J16.3, RP11277P12.20, MS4A6A, C9orf66, TRIO, PRKAR2B, CYP24A1, KLRK1..4, MMP25, TST, CTD2325P2.4, MAN1A1, RPPH1, PPFIBP1, KLRK1..6, UBE2E2, SORCS1, KIAA1456..1, COL24A1, LRP1, TRAIP, GPR97, FGD6, LMLN, EHHADH, ZNF461, CSF2, TRPM8, MCAM, CD38, FAM167A, C1orf159, OSBPL6 | ^2^ |
| IL17a | NFKBIZ,ZC3H12A,RELB,BIRC3,CXCL1,ZC3H12C,TNFAIP3,MAP3K8,CXCL2,CASZ1,IER3,LENG1,SOD2,RRAD,NFKB2,ID2,TATDN1,CBR3,CCL3,ICAM1,PIK3R6,GADD45B,ADAM21,ZBTB25 | ^8^ |
| Th2 activated | ABCA2, ACTN1, ASPH, BAI2, BCL2L11, BEGAIN, BEST4, BTBD11, C16orf45, C6orf105, CADM1, CAPG, CCR7, CD27, DUSP23, E2F3, FAM55C, FANK1, FLJ32810, FLJ45983, FLJ46446, GAB2, GALT, GATA3, GBP5, GPA33, HOMER3, IGF1R, ITPRIPL1, KBTBD11, KLRB1, LMCD1, LOC100128031, LOC100131176, LOC729041, MBD5, METRNL, MRC2, NECAB3, NEFL, NOD2, NOS3, NRIP1, NRIP3, PELI2, PPAPDC1B, PPP1R9B, PRAGMIN, PRMT10, RORC, SEMA5A, SLC12A6, SLC25A33, SNED1, SNN, SNTN, SUSD4, TMEM2, TMIGD2, TNFRSF18, TPPP, TULP4, TXK, ZBTB16, ZDHHC11, ZDHHC11B, ZP1 | ^9^ |
| Treg activated | ACSL6, ANK3, AP3M2, APP, ASPH, AXIN2, BASP1, BFSP2, C1orf228, CACNA1I, CD40LG, CD70, CKS2, CPNE2, CTLA4, CTNNAL1, CXCR5, DENND5A, DPP4, DUSP10, DUSP4, F5, FANK1, FLJ46446, FOXP3, GBP5, GPR35, HLA-DMA, HLA-DMB, HLA-DOA, HLA-DRB1, HLA-DRB4, HS3ST3B1, ICA1, ID2, IKZF2, IKZF4, IL2RA, IL7R, IRF4, KLRB1, LMCD1, LOC100131646, LOC100132345, LOC284475, LOC541471, LOC643988, LOC650392, LOC730631, MGST2, MSX2P1, NELL2, NLRP3, NOG, NOSIP, NR3C2, NUSAP1, PCTP, PDIA5, PMAIP1, PTPLA, RBMS1, RECK, RHOXF1, RORC, RP3-527F8.2, RPL39L, SCML4, SETD7, SHMT2, SLC2A8, SLC40A1, SMC6, SMPD3, SOS2, SOX8, SPATS2L, STAT4, THEMIS, TIGIT, TMEM71, TNFRSF1B, TOX, TRIB1, TRIM16L, TSPAN18, VAV3, VIPR1, WNT10B, WNT7A, WNT7B, ZFP36L2 | ^9^ |
| CD4 | ALOX5, ANXA4, BIVM, BPI, BRD7, CAMP, CASK, CD63, CEACAM6, CEACAM8, CFLAR, DEFA4, GCA, LCN2, LOC23117, LOC728358, LTF, MBOAT1, MMP8, MNDA, PDE4D, POLR2J2, PPP1R16B, PRKCB1, RETN, RNASE3, RUNX2, S100A12, S100A8, S100A9, S100P, SLC38A2, SPATA13, SRGN, SRGN, STOM, TNIP1, TTYH2, UGCG, ZNF749 | ^10^ |
| Eosinophils | CLC, SIGLEC8, EMR1, EMR4P, LGAL312, HRH4, CEBPE, DACH1, VSTM1 | ^11^ |
| Mast cell | MS4A2, TPSAB1, TPSAB2, CPA3, RGS13, C1orf186 | ^11^ |
| Macrophage GM-CSF.IL4 | CBR3, CLEC10A, FZD2, CD1C, SIGLEC10, PARM1, CCL26, FCER2, MAOA, MAP4K1, FOXQ1, HOMER2, PDXK, GALNT18, NAGPA, CD209, INSIG2, RTKN, SUCNR1, NFXL1, FAM60A, FCER1A, DUOX1, ASAP1, CXCR2P1, FGL2, GPD1L, PALLD, QPRT, C17orf58, C1orf54, CMTM8, CARD9, ADAM19, LIPA, SLC27A3, CTNNAL1, ASCL2, CD1A, RAB33A, DHRS2, RASGRP3, GATM, DTNA, RASSF7, ZNF366, BLOC1S6, PTPRE, ACOT7, STARD7, CCDC6, RASL10A, INTS3, ST6GAL1, DHRS11, ADAM15, PTRF, SGK223 ,PPP1R14A, BATF3, RAMP1, CERK, MMP12, SLAMF1, IL1R2, KIAA1671, SOCS1, RAP1GAP, ALOX15, CD1E, CDKN1A, SOX8, DENND1B, VCL, SPINT2, PFKP, RHBDF1, FSCN1, RRP1B, SNX8, LOC283070, PPM1F, GOLGA8B, ESPNL, CDR2L, NMNAT3, CCL22, TTC9C, CLEC4G, AUH, PTPRO, CLEC4A, CHN2 | ^6^ |
| Macrophage GM-CSF.TNFa. HS.IVS.UP | ZFYVE16, HNRNPDL, MCTP1, SLC7A11, IL1A, MCOLN2, NEK6, DDIT4, MAOA, NUB1, PHLDB1, TNS3, DRAM1, COX10, SLC20A1, VNN1, INSIG2, OCSTAMP, PTPN1, FAM60A, IL2RA, SLC7A1, ETS1, RHBDF2, G0S2, TSC22D1, EEF1B2, IDH3A, C17orf58, TRAF3IP2, PACSIN2, LMO4, TNIP1, MPO, ACSL5, CS, KLHL5, BTG3, OTUD4, PLGRKT, F5, SIAH1, NFKB1, AQP9, LAMB3, RPL32, ADAMDEC1, PFDN1, SYT11, MT1F, TNC, SLC7A5, CA12, METTL21B, MRPS6, GK, BHLHE41, WARS, EFTUD1, SRC, CCDC115, FILIP1L, ARFGAP3, ATP1A1, PTPRE, NBN, HIVEP1, GRAMD1A, KYNU, SLC1A3, NFE2L1, PCNX, CYP27B1, ANXA5, QPCT, SH3PXD2A, SERPINE1, CTTN, CLINT1, TTC13, IRAK2, SLC41A2, TSPAN33, ACO1, RNF144B, HNRNPA0, FAM213A, NFS1, MTF1, BCAT1, UCK2, SLAMF1, STRN3, GPR124, CCL5, IL1B, BIRC3, SLC50A1, SLC2A6, CLEC5A, LINC00938, PRKCH, LAD1, SLC25A3, GPR84, DENND5A, TNFAIP6, SLC35F2, CRISPLD2, PRKCDBP, DNAJB6, NDP, PLEK, MMP9, SPARC, F3, ANKRD22, SLC9A1, RFTN1, TSKU, EBI3, PPA1, ADORA2A, RGS16, MYOF, ATP5L, METTL1, TNPO1, AK4, ESYT2, SCG5, COA1, FNBP4, TNFRSF4, HBEGF, MT1H, TMEM194A, ZSWIM4, IL36G, RPS4X, ACVR1B, BCL2, FYN, DTX2, HOPX, TMEM132A, PTGES2, EMR2, SPHK1, SFXN4, BID, VEGFA, PTPRF, C1QTNF1, HSD11B1, C6orf48, CD82, FMNL3, MTHFD2, SOD2 | ^6^ |

**References**

1.	Alevy YG, Patel AC, Romero AG, et al. IL-13-induced airway mucus production is attenuated by MAPK13 inhibition. *J Clin Invest.* 2012;122:4555-4568.

2.	Bjorklund AK, Forkel M, Picelli S, et al. The heterogeneity of human CD127(+) innate lymphoid cells revealed by single-cell RNA sequencing. *Nat Immunol.* 2016;17:451-460.

3.	Simpson JL, Phipps S, Baines KJ, Oreo KM, Gunawardhana L, Gibson PG. Elevated expression of the NLRP3 inflammasome in neutrophilic asthma. *Eur Respir J.* 2014;43:1067-1076.

4.	Liberzon A, Subramanian A, Pinchback R, Thorvaldsdottir H, Tamayo P, Mesirov JP. Molecular signatures database (MSigDB) 3.0. *Bioinformatics.* 2011;27:1739-1740.

5.	Abbas AR, Baldwin D, Ma Y, et al. Immune response in silico (IRIS): immune-specific genes identified from a compendium of microarray expression data. *Genes Immun.* 2005;6:319-331.

6.	Xue J, Schmidt SV, Sander J, et al. Transcriptome-based network analysis reveals a spectrum model of human macrophage activation. *Immunity.* 2014;40:274-288.

7.	Zhang H, Nestor CE, Zhao S, et al. Profiling of human CD4+ T-cell subsets identifies the TH2-specific noncoding RNA GATA3-AS1. *J Allergy Clin Immunol.* 2013;132:1005-1008.

8.	Dragon S, Hirst SJ, Lee TH, Gounni AS. IL-17A mediates a selective gene expression profile in asthmatic human airway smooth muscle cells. *American journal of respiratory cell and molecular biology.* 2014;50:1053-1063.

9.	Zhang H, Nestor CE, Zhao S, et al. Profiling of human CD4+ T-cell subsets identifies the TH2-specific noncoding RNA GATA3-AS1. *Journal of Allergy and Clinical Immunology.* 2013;132:1005-1008.

10.	Tsitsiou E, Williams AE, Moschos SA, et al. Transcriptome analysis shows activation of circulating CD8+ T cells in patients with severe asthma. *Journal of Allergy and Clinical Immunology.* 2012;129:95-103.

11.	Leaker BR, Malkov VA, Mogg R, et al. The nasal mucosal late allergic reaction to grass pollen involves type 2 inflammation (IL-5 and IL-13), the inflammasome (IL-1β), and complement. *Mucosal immunology.* 2017;10:408-420.
