# Supplementary material for: Clinical Features of Cellular Senescence Pathways in Severe Asthma
Source: Allergy. 2026 Jun 18;81(9):3273–85. doi: 10.1111/all.70416 (PMC13569611; doi:10.1111/all.70416)
Supplement: Supplementary file 5 [file ALL-81-3273-s005.docx]

**Table S5. Comparison of clinical and demographic characteristics according to the p53/SASP quadrant classification**

|  | p53-low/SASP-low | p53-low/SASP-high | p53-high/SASP-low | p53-high/SASP-high | *p* value |
| --- | --- | --- | --- | --- | --- |
| Participant, n | 33 | 11 | 6 | 30 |  |
| Age (years) | 49.0 [35.0–57.0] | 52.0 [34.5–59.5] | 42.5 [38.0–48.5] | 49.0 [36.5–58.0] | 0.846 |
| Female, n (%) | 13 (39) | 5 (45) | 3 (50) | 16 (53) | 0.737 |
| BMI (kg/m^2^) | 28.3 ± 5.5 | 29.9 ± 4.3 | 27.7 ± 8.6 | 28.1 ± 6.2 | 0.505 |
| Smoking status, n (%) |  |  |  |  | 0.717 |
| Current smoker | 2 (6) | 1 (9) | 1 (17) | 3 (10) |  |
| Former smoker | 6 (18) | 4 (36) | 1 (17) | 4 (13) |  |
| Never smoker | 25 (76) | 6 (55) | 4 (67) | 23 (77) |  |
| Smoking (pack-years) | 0.0 [0.0–0.0] | 0.0 [0.0–11.8] | 0.0 [0.0–3.8] | 0.0 [0.0–0.0] | 0.502 |
| Asthma onset age (years) | 10.0 [3.5–29.5] | 36.0 [10.0–48.8] | 14.5 [7.0–26.5] | 11.0 [3.2–39.2] | 0.393 |
| Asthma duration (years) | 25.0 [20.0–42.5] | 13.5 [6.2–22.8] | 25.5 [16.5–36.0] | 25.5 [17.0–36.8] | 0.233 |
| Allergic rhinitis, n (%) | 18 (55) | 6 (55) | 6 (100) | 17 (59) | 0.205 |
| Nasal polyps, n (%) | 2 (6) | 2 (18) | 2 (33) | 13 (45) | 0.006 |
| Maintenance OCS use, n (%) | 5 (15) | 4 (36) | 3 (50) | 14 (47) | 0.042 |
| Exacerbation frequency in the previous year | 1.0 [1.0–2.0] | 3.0 [1.0–9.5] | 2.0 [2.0–8.8] | 9.0 [1.2–11.0] | 0.008 |
| FEV1% pred (%) | 88.7 [68.1–97.5] | 74.1 [64.8–96.7] | 84.3 [72.6–97.9] | 73.8 [51.6–87.6] | 0.093 |
| FVC% pred (%) | 102.4 [87.9–111.4] | 99.5 [85.6–110.5] | 99.9 [91.0–110.3] | 90.3 [78.3–99.7] | 0.131 |
| FEV1/FVC ratio (%) | 70.7 [65.1–76.7] | 67.6 [52.9–78.5] | 71.3 [64.3–78.7] | 66.4 [55.6–76.6] | 0.429 |
| Post-BD FEV1% pred (%) | 92.9 [78.7–108.2] | 82.0 [74.6–109.3] | 91.7 [81.5–103.0] | 79.8 [56.9–94.0] | 0.028 |
| FeNO (ppb) | 21.0 [13.6–41.8] | 21.0 [17.5–38.5] | 25.0 [10.6–33.0] | 23.5 [17.2–53.0] | 0.740 |
| Total IgE (IU/mL) | 86.8 [44.8–172.8] | 129.0 [20.5–358.0] | 118.5 [77.9–270.0] | 132.5 [31.7–368.5] | 0.993 |
| Serum periostin (ng/mL) | 43.5 [39.0–47.8] | 43.8 [39.9–50.3] | 48.9 [40.4–59.9] | 46.9 [41.6–57.6] | 0.258 |
| Blood eosinophil (x 10^9^/L) | 0.2 [0.1–0.3] | 0.2 [0.1–0.3] | 0.3 [0.1–0.3] | 0.2 [0.1–0.3] | 0.883 |
| Blood neutrophil (x 10^9^/L) | 4.6 [3.0–5.5] | 3.8 [3.5–5.0] | 4.4 [3.6–5.2] | 4.3 [3.3–6.1] | 0.916 |

Data are presented as median [IQR] for continuous variables (mean ± SD for BMI) and n (%) for categorical variables. *P* values are derived from the Kruskal–Wallis test for continuous variables and the χ² test for categorical variables across the four quadrants.

Abbreviations: SASP, senescence-associated secretory phenotype; OCS, oral corticosteroids; FEV1, forced expiratory volume in 1 second; FVC, forced vital capacity; BD, bronchodilator; FeNO, fractional exhaled nitric oxide; IgE, immunoglobulin E.
