# Supplementary material for: Clinical Features of Cellular Senescence Pathways in Severe Asthma
Source: Allergy. 2026 Jun 18;81(9):3273–85. doi: 10.1111/all.70416 (PMC13569611; doi:10.1111/all.70416)
Supplement: Supplementary file 6 [file ALL-81-3273-s006.docx]

**Table S1. Cellular senescence-related signatures used for the gene set variation analyses**

| Name of signature | Genes | Reference |
| --- | --- | --- |
| p53 pathway | ADGRB1 APAF1 ATM ATR BAX BBC3 BID CASP3 CASP8 CASP9 CCNB1 CCNB2 CCNB3 CCND1 CCND2 CCND3 CCNE1 CCNE2 CCNG1 CCNG2 CD82 CDK1 CDK2 CDK4 CDK6 CDKN1A CDKN2A CHEK1 CHEK2 COP1 CYCS DDB2 EI24 FAS GADD45A GADD45B GADD45G GTSE1 IGF1 IGFBP3 MDM2 MDM4 PERP PIDD1 PMAIP1 PPM1D PTEN RCHY1 RPRM RRM2 RRM2B SERPINB5 SERPINE1 SESN1 SESN2 SESN3 SFN SHISA5 SIAH1 STEAP3 THBS1 TNFRSF10B TP53 TP53AIP1 TP53I3 TP73 TSC2 ZMAT3 | hsa04115  p53 signaling pathway  Homo sapiens (human) |
| SASP | ANAPC1 ANAPC10 ANAPC11 ANAPC15 ANAPC16 ANAPC2 ANAPC4 ANAPC5 ANAPC7 CCNA1 CCNA2 CDC16 CDC23 CDC26 CDC27 CDK2 CDK4 CDK6 CDKN1A CDKN1B CDKN2A CDKN2B CDKN2C CDKN2D CEBPB CXCL8 EHMT1 EHMT2 FOS FZR1 H2AFB1 H2AFJ H2AFV H2AFX H2AFZ H2BFS H3F3A H3F3B HIST1H2AB HIST1H2AC HIST1H2AD HIST1H2AE HIST1H2AJ HIST1H2BA HIST1H2BB HIST1H2BC HIST1H2BD HIST1H2BE HIST1H2BF HIST1H2BG HIST1H2BH HIST1H2BI HIST1H2BJ HIST1H2BK HIST1H2BL HIST1H2BM HIST1H2BN HIST1H2BO HIST1H3A HIST1H3D HIST1H3E HIST1H3F HIST1H3G HIST1H3H HIST1H3I HIST1H3J HIST1H4A HIST1H4B HIST1H4C HIST1H4D HIST1H4E HIST1H4F HIST1H4H HIST1H4I HIST1H4J HIST1H4K HIST1H4L HIST2H2AA3 HIST2H2AA4 HIST2H2AC HIST2H2BE HIST2H3A HIST2H3C HIST2H3D HIST2H4A HIST2H4B HIST3H2BB HIST4H4 IGFBP7 IL1A IL6 JUN MAPK1 MAPK3 MAPK7 NFKB1 RELA RPS27A RPS6KA1 RPS6KA2 RPS6KA3 STAT3 UBA52 UBB UBC UBE2C UBE2D1 UBE2E1 VENTX | R-HSA-2559582  Senescence-Associated Secretory Phenotype (SASP)  Homo sapiens (human) |
