# Supplementary material for: Clinical Features of Cellular Senescence Pathways in Severe Asthma
Source: Allergy. 2026 Jun 18;81(9):3273–85. doi: 10.1111/all.70416 (PMC13569611; doi:10.1111/all.70416)
Supplement: Supplementary file 7 [file ALL-81-3273-s007.docx]

**Table S4. Multivariate linear regression between clinical parameters and senescence enrichment scores in bronchial biopsies of asthma patients (n=81)**

| **Bronchial biopsy** | **p53 signalling pathway gene set ES** | | **SASP gene set ES** | |
| --- | --- | --- | --- | --- |
| Parameter | Regression coefficient (95% CI)† | *P* value | Regression coefficient (95% CI)† | *P* value |
| Exacerbations/year* | 0.068 (0.022 to 0.114) | 0.005 | 0.086 (0.038 to 0.134) | <0.001 |
| Nasal polyp (vs. no) | 0.208 (0.084 to 0.333) | 0.010 | 0.151 (0.013 to 0.288) | 0.033 |
| Post-BD FEV1% pred (%) | -0.269 (-0.473 to -0.066) | 0.012 | -0.270 (-0.487 to -0.054) | 0.015 |
| Serum periostin (ng/ml)* | 0.309 (0.071 to 0.546) | 0.012 | 0.267 (0.003 to 0.531) | 0.048 |

ES, enrichment score; BD, bronchodilator; FEV1, forced expiratory volume in 1 second; SASP, senescence-associated secretory phenotype.

Adjusted for age, sex, BMI, smoking status, and daily maintenance OCS use

*Variable log-transformed to normalize distribution.
